# Multidisciplinary management of elderly patients with rectal cancer: recommendations from the SICG (Italian Society of Geriatric Surgery), SIFIPAC (Italian Society of Surgical Pathophysiology), SICE (Italian Society of Endoscopic Surgery and new technologies), and the WSES (World Society of Emergency Surgery) International Consensus Project

**DOI:** 10.1186/s13017-021-00378-9

**Published:** 2021-07-02

**Authors:** Mauro Podda, Patricia Sylla, Gianluca Baiocchi, Michel Adamina, Vanni Agnoletti, Ferdinando Agresta, Luca Ansaloni, Alberto Arezzo, Nicola Avenia, Walter Biffl, Antonio Biondi, Simona Bui, Fabio C. Campanile, Paolo Carcoforo, Claudia Commisso, Antonio Crucitti, Nicola De’Angelis, Gian Luigi De’Angelis, Massimo De Filippo, Belinda De Simone, Salomone Di Saverio, Giorgio Ercolani, Gustavo P. Fraga, Francesco Gabrielli, Federica Gaiani, Mario Guerrieri, Angelo Guttadauro, Yoram Kluger, Ari K. Leppaniemi, Andrea Loffredo, Tiziana Meschi, Ernest E. Moore, Monica Ortenzi, Francesco Pata, Dario Parini, Adolfo Pisanu, Gilberto Poggioli, Andrea Polistena, Alessandro Puzziello, Fabio Rondelli, Massimo Sartelli, Neil Smart, Michael E. Sugrue, Patricia Tejedor, Marco Vacante, Federico Coccolini, Justin Davies, Fausto Catena

**Affiliations:** 1 Department of Emergency Surgery, Cagliari University Hospital “D. Casula”, Azienda Ospedaliero-Universitaria di Cagliari, Cagliari, Italy; 2 Division of Colon and Rectal Surgery, Mount Sinai Hospital, New York, NY USA; 3 ASST Cremona, Department of Clinical and Experimental Sciences, University of Brescia, Brescia, Italy; 4 Department of Colorectal Surgery, Cantonal Hospital of Winterthur, Winterthur - University of Basel, Basel, Switzerland; 5 ICU Department, Bufalini Hospital, Cesena, Italy; 6 Department of General Surgery, Vittorio Veneto Hospital, AULSS2 Trevigiana del Veneto, Vittorio Veneto, Italy; 7 1st General Surgery Unit, University of Pavia, Pavia, Italy; 8 Department of Surgical Sciences, University of Torino, Torino, Italy; 9 SC Chirurgia Generale e Specialità Chirurgiche Azienda Ospedaliera Santa Maria, Università degli Studi di Perugia, Terni, Italy; 10 Trauma and Acute Care Surgery, Scripps Memorial Hospital, La Jolla, CA USA; 11 Department of General Surgery and Medical - Surgical Specialties, University of Catania, Catania, Italy; 12 Department of Medical Oncology, Azienda Ospedaliero-Universitaria di Parma, Parma, Italy; 13 Department of Surgery, ASL VT - Ospedale “San Giovanni Decollato - Andosilla”, Civita Castellana, Italy; 14 Department of Surgery, Unit of General Surgery, University Hospital of Ferrara, University of Ferrara, Ferrara, Italy; 15 Department of Radiology, University Hospital of Parma, Parma, Italy; 16 General and Minimally Invasive Surgery Unit, Cristo Re Hospital and Catholic University, Rome, Italy; 17 Unit of Minimally Invasive and Robotic Digestive Surgery, Regional General Hospital F. Miulli, Acquaviva delle Fonti, Bari, Italy; 18 Department of Medicine and Surgery, Gastroenterology and Endoscopy Unit, University of Parma, Parma, Italy; 19 Department of General and Metabolic Surgery, Poissy and Saint Germain en Laye Hospitals, Poissy, France; 20 Department of Surgery, University of Insubria, Varese, Italy; 21 General and Oncologic Surgery, Morgagni-Pierantoni Hospital, AUSL Romagna, Forlì, Italy; 22 Division of Trauma Surgery, School of Medical Sciences, University of Campinas, Campinas, SP Brazil; 23 University of Milano Bicocca, Milano, Italy; 24 Università Politecnica delle Marche, Ancona, Italy; 25 Division of General Surgery, Rambam Health Care Campus, Haifa, Israel; 26 Helsinki University Hospital, University of Helsinki, Helsinki, Finland; 27 UOC Chirurgia Generale - AOU san Giovanni di Dio e Ruggi d’Aragona, Università di Salerno, Salerno, Italy; 28 Department of Medicine and Surgery, University of Parma Geriatric-Rehabilitation Department, Parma University Hospital, Parma, Italy; 29 Ernest E Moore Shock Trauma Center at Denver Health, Denver, USA; 30 Ospedale Nicola Giannettasio, Corigliano-Rossano, Italy; 31 Santa Maria della Misericordia Hospital, Rovigo, Italy; 32 Surgery of the Alimentary Tract, Sant’Orsola Hospital, Department of Medical and Surgical Sciences, Alma Mater Studiorum University of Bologna, Bologna, Italy; 33 Dipartimento di Chirurgia Pietro Valdoni Policlinico Umberto I, Sapienza Università degli Studi di Roma, Rome, Italy; 34 Department of Surgery, Macerata Hospital, Macerata, Italy; 35 Exeter Hospital, Exeter, UK; 36 Letterkenny University Hospital and CPM sEUBP Interreg Project, Letterkenny, Ireland; 37 University Hospital ‘Gregorio Marañón’, Madrid, Spain; 38 General, Emergency and Trauma Surgery Department, Pisa University Hospital, Pisa, Italy; 39 Cambridge Colorectal Unit, Addenbrooke’s Hospital, Cambridge, UK; 40 Department of Emergency Surgery, Parma Maggiore Hospital, Parma, Italy

**Keywords:** Rectal cancer, Elderly, Frailty, Multidisciplinary management, Consensus

## Abstract

**Background and aims:**

Although rectal cancer is predominantly a disease of older patients, current guidelines do not incorporate optimal treatment recommendations for the elderly and address only partially the associated specific challenges encountered in this population. This results in a wide variation and disparity in delivering a standard of care to this subset of patients. As the burden of rectal cancer in the elderly population continues to increase, it is crucial to assess whether current recommendations on treatment strategies for the general population can be adopted for the older adults, with the same beneficial oncological and functional outcomes. This multidisciplinary experts’ consensus aims to refine current rectal cancer-specific guidelines for the elderly population in order to help to maximize rectal cancer therapeutic strategies while minimizing adverse impacts on functional outcomes and quality of life for these patients.

**Methods:**

The discussion among the steering group of clinical experts and methodologists from the societies’ expert panel involved clinicians practicing in general surgery, colorectal surgery, surgical oncology, geriatric oncology, geriatrics, gastroenterologists, radiologists, oncologists, radiation oncologists, and endoscopists. Research topics and questions were formulated, revised, and unanimously approved by all experts in two subsequent modified Delphi rounds in December 2020–January 2021. The steering committee was divided into nine teams following the main research field of members. Each conducted their literature search and drafted statements and recommendations on their research question.

Literature search has been updated up to 2020 and statements and recommendations have been developed according to the GRADE methodology. A modified Delphi methodology was implemented to reach agreement among the experts on all statements and recommendations.

**Conclusions:**

The 2021 SICG-SIFIPAC-SICE-WSES consensus for the multidisciplinary management of elderly patients with rectal cancer aims to provide updated evidence-based statements and recommendations on each of the following topics: epidemiology, pre-intervention strategies, diagnosis and staging, neoadjuvant chemoradiation, surgery, watch and wait strategy, adjuvant chemotherapy, synchronous liver metastases, and emergency presentation of rectal cancer.

## Introduction

According to the World Health Organization, colorectal cancer is the third most commonly diagnosed cancer in males and the second in females. Although rectal cancer, with a mean age at the time of diagnosis of 68 years for men and 72 years for women, is predominantly a disease of older patients, [1], current guidelines do not incorporate optimal treatment recommendations for the elderly and address only partially the associated specific challenges encountered in this population. This results in a wide variation and disparity in delivering a standard of care to this subset of patients [2]. With the aging population, the number of elderly rectal cancer patients is expected to increase further. These patients often have more comorbidities, increased complication rates, and a poorer prognosis [3].

There is a paucity of clinical trial evidence explicitly addressing the risks and benefits of all aspects of rectal cancer in the elderly, which is mainly attributable to the fact that older adults comprise a heterogenous population covering a spectrum from very frail to fit patients. Moreover, although surgeons are usually accustomed to operating on elderly patients, both in emergency and elective settings [4], recent data indicate that older adults affected by rectal cancer are more likely to be offered a suboptimal range of care for their disease.

The EURECCA international study on the treatment and survival of rectal cancer patients over the age of 80 years in Belgium, Denmark, the Netherlands, Norway, and Sweden found a substantial variation in the 5-year relative survival between European countries, next to a wide variation in treatment modalities, especially in the use of preoperative radiotherapy in stage II–III patients and the rate of stage IV patients undergoing surgery. Overall, among over 19.500 rectal cancer patients included, 5-year relative survival varied from 61.7% in Belgium to 72.3% in Sweden for stage I–III patients. The proportion receiving preoperative radiotherapy ranged between 7.9% in Norway and 28.9% in Sweden, whereas the rate of patients undergoing surgery varied from 22.2% in Denmark to 40.8% in Norway. An explanation for the lower use of preoperative radiotherapy in elderly patients with rectal cancer might be that a higher risk of recurrence may be considered acceptable in these patients, as in this group maintaining function and quality of life is of great importance [5, 6].

Treatment regimens for rectal cancer patients are more challenging to tolerate than colon cancer, especially for old frail patients. The current standard of care for stage II and III rectal cancer requires multimodality treatments that include three phases: neoadjuvant chemo-radiotherapy (nCRT), surgery with rectal resection, and postoperative adjuvant chemotherapy. In the study by Thiels et al., a total of 160 elderly patients (median age 80 years) with stage II and stage III rectal cancer underwent surgical resection. However, only 30% and 33.8% received neoadjuvant or adjuvant therapy, respectively. Among patients with stage II rectal cancer, there was no significant difference in 60-month survival between patients who received any additional therapy and those who had surgery alone. Conversely, additional therapy (neoadjuvant chemotherapy or chemo-radiotherapy or adjuvant chemotherapy) improved survival in patients with stage III tumors (58% vs. 30%) [7].

As the older rectal cancer patients’ prognosis and treatment decisions are primarily influenced by comorbidity and frailty, there is a growing awareness of the need for geriatric assessment as an essential component in the preoperative workup.

Many oncology and surgical societies agree that frail and vulnerable patients could access standard treatments after being adequately pre-habilitated. For this reason, they have established specific task forces to include in their guidelines recommendations that provide an in-depth analysis of all domains of functioning (functional, physical, mental, emotional, pharmacological, and socioeconomic) that can help in determining potential compliance of intensive anti-cancer treatments in the elderly [8]. The interest is justified, as it has been recently pointed out that for the fitter elderly, the multimodal treatment including nCRT, resectional surgery, and adjuvant chemotherapy leads to cancer-specific survival rates comparable to those found in the younger population [9]. On the other hand, for patients at higher risk of toxicity or those who refuse surgery, response to neoadjuvant treatment is emerging as a new prognostic factor.

Areas of major debate in the treatment of elderly patients with rectal cancer also remain about the watch and wait strategies after neoadjuvant therapy [10], local excision [11], fractionation and duration of radiotherapy (short course vs. long course), the optimal time to surgery [12], and the benefit of adjuvant chemotherapy.

Cancer treatment in the elderly is also different from the general population in terms of outcomes prioritization and goals of the whole therapeutic strategy. Tailored therapies, including surgical interventions, should focus on the patients’ quality of life and functional recovery rather than simple conventional 5-year disease-free survival while maintaining, as far as possible, oncological standards.

The surgical population has increased not only in volume, but also in comorbidity profile and age, requiring an improved preoperative selection and definition of classes of risk for surgery and antineoplastic therapies.

The questions arising from the debates on the management of cancer in the older population problem are multiple: Is the patient going to die with cancer or of cancer? Is the patient able to tolerate the stress of chemotherapy? Is the treatment producing more benefits than harm?

A multidimensional geriatric assessment can identify three different categories of patients based on their life expectancy and functional status: “fit patients” (people who are functionally independent and without comorbidity) who are candidates for any form of standard cancer treatment and can receive the same treatments as younger patients; “vulnerable patients” who require tailored treatment strategies with some special pharmacological schemes, such as reduction in the initial dose of chemotherapy with subsequent dose escalations, or surgical strategy; and “frail patients” (dependence in one or more activities of daily living, three or more comorbid conditions, one or more geriatric syndromes) who are only candidates for palliative treatment [13, 14].

Since elderly patients older than 70 have been excluded from randomized controlled trials (RCT), very little evidence exists in this population, most of it not being of high level [15]. The project of this experts’ consensus arises from the acknowledgment of the lack of evidence about the subgroup of elderly patients with rectal cancer. As the burden of rectal cancer in the elderly population continues to increase, it is crucial to assess whether current recommendations on treatment strategies for the general population can be adopted for the older adults, with the same beneficial oncological and functional outcomes. This experts’ consensus aims to refine current rectal cancer-specific guidelines for the elderly population in order to help to maximize rectal cancer therapeutic strategies while minimizing adverse impacts on functional outcomes and quality of life for these patients. Evidence in the present consensus document has been summarized taking into account the different baseline health conditions of elderly rectal cancer patients, although grouping of patients into surgically fit and unfit categories remains largely subjective.

## Methods

This consensus document has been created by a multisocietary collaboration between the SICG (Società Italiana di Chirurgia Geriatrica—Italian Society of Geriatric Surgery), the SIFIPAC (Società Italiana di Fisiopatologia Chirurgica—Italian Society of Surgical Physiopathology), the SICE (Società Italiana di Chirurgia Endoscopica e nuove tecnologie—Italian Society of Endoscopic Surgery and new technologies) and the WSES.It (World Society of Emergency Surgery—Italy Chapter). The discussion among the steering group of clinical experts and methodologists from the societies’ expert panel involved clinicians practicing in general surgery, colorectal surgery, surgical oncology, geriatric oncology, geriatrics, gastroenterologists, radiologists, oncologists, radiation oncologists, and endoscopists.

### Topic elaboration and prioritization

The subject of rectal cancer in the elderly (≥ 70 years old) was divided into nine main topics: epidemiology (socioeconomic burden, screening strategies), pre-intervention strategies (improvement strategies for patient involvement in healthcare decision-making, frailty assessment and multidisciplinary evaluation, definition, and prioritization of patient-centered outcomes), diagnosis and staging, neoadjuvant chemoradiation (indication, timing, compliance, and outcomes of neoadjuvant chemoradiation), surgery (prehabilitation, enhanced recovery after surgery, oral antibiotic prophylaxis, local excision, minimally invasive surgery with laparoscopic/robotic TME and TaTME, early versus delayed ileostomy closure) watch and wait (indications and outcomes), adjuvant chemotherapy (indications and outcomes), liver disease (treatment of synchronous liver metastases) and emergency presentation (obstructing rectal cancer).

Research topics and questions were formulated, revised, and unanimously approved by all experts in two subsequent modified Delphi rounds in December 2020–January 2021. The steering committee was divided into nine teams following the main research field of members. Each conducted their literature search and drafted statements and recommendations on their research question.

### Literature review

Based on the research questions, the literature review process was carried out conforming to the PRISMA statement standards for systematic reviews and meta-analyses [16] between December 22th 2020, and February 28th, 2021. MEDLINE (via PubMed), the Cochrane Central Register of Controlled Trials, and EMBASE were systematically searched for relevant studies. Study inclusion criteria included systematic reviews with or without meta-analyses, randomized controlled trials, and non-randomized cohort studies on the subject of rectal cancer in elderly patients (≥ 70 years old) published in the English language without any restriction of publication date. Animal studies, case reports, narrative reviews, commentaries, and studies on colorectal cancer not including specific information on rectal localization of the cancer were excluded.

### The GRADE methodology

The statements were formulated and graded according to the Grading of Recommendations Assessment, Development and Evaluation (GRADE) hierarchy of evidence [17], summarized in Table 1. The quality of evidence (QoE) was marked as high, moderate, low, or very low. This could be either downgraded in case of significant bias or upgraded when multiple high-quality studies showed consistent results. The highest quality of evidence studies (systematic reviews with meta-analysis of randomized controlled trials) was assessed first. If the meta-analyses were of sufficient quality, they were used to answer the research question. If no meta-analysis of sufficient quality was found, randomized controlled trials (RCTs) and non-randomized cohort studies (n-RCS) were evaluated. The strength of the recommendation (SoR) was based on the level of evidence and qualified as weak or strong [18, 19]. Statements and recommendations were generated in response to each research question based on the literature review, using GRADE criteria for assigning strength. The content and strength of each statement and recommendation were reviewed by the panel group’s systematic review team, taking into account the quality of the supporting evidence.

**Table 1:** GRADE quality of evidence and strength of recommendations

Quality of evidence and strength of recommendation	Clarity of balance between desirable and undesirable effects	Methodological quality of supporting evidence	Implications
**High-quality evidence, strong recommendation**	1.1.1.1.1.1.1.1.1. Desirable effects clearly outweigh undesirable effects, or vice versa	1.1.1.1.1.1.1.1.2. Consistent evidence from well-performed RCTs or exceptionally strong evidence from unbiased observational studies	1.1.1.1.1.1.1.1.3. Recommendation can apply to most patients in most circumstances. Further research is unlikely to change our confidence in the estimate effect
**Moderate quality evidence, strong recommendation**	1.1.1.1.1.1.1.1.4. Desirable effects clearly outweigh undesirable effects, or vice versa	1.1.1.1.1.1.1.1.5. Evidence from RCTs with important limitations (inconsistent results, methodological flaws, indirectness, imprecision) or exceptionally strong evidence from unbiased observational studies	1.1.1.1.1.1.1.1.6. Recommendation can apply to most patients in most circumstances. Further research (if performed) is likely to have an important impact on our confidence in the estimate of effect and may change the estimate
**Low-quality evidence, strong recommendation**	1.1.1.1.1.1.1.1.7. Desirable effects clearly outweigh undesirable effects, or vice versa	1.1.1.1.1.1.1.1.8. Evidence for at least one critical outcome from observational studies, RCTs with serious flaws or indirect evidence	1.1.1.1.1.1.1.1.9. Recommendation may change when higher quality evidence becomes available. Further research (if performed) is likely to have an important impact on our confidence in the estimate of effect and is likely to change the estimate
**Very low-quality evidence, strong recommendation (rarely applicable)**	1.1.1.1.1.1.1.1.10. Desirable effects clearly outweigh undesirable effects, or vice versa	1.1.1.1.1.1.1.1.11. Evidence for at least one critical outcome from unsystematic clinical observations or very indirect evidence	1.1.1.1.1.1.1.1.12. Recommendation may change when higher quality evidence becomes available; any estimate of effect for at least one critical outcome is very uncertain
**High-quality evidence, weak recommendation**	1.1.1.1.1.1.1.1.13. Desirable effects closely balanced with undesirable effects	1.1.1.1.1.1.1.1.14. Consistent evidence from well-performed RCTs or exceptionally strong evidence from unbiased observational studies	1.1.1.1.1.1.1.1.15. The best action may differ depending on circumstances or patients or societal values. Further research is unlikely to change our confidence in the estimate effect
**Moderate-quality evidence, weak recommendation**	1.1.1.1.1.1.1.1.16. Desirable effects closely balanced with undesirable effects	1.1.1.1.1.1.1.1.17. Evidence from RCTs with important limitations (inconsistent results, methodological flaws, indirectness, imprecision) or exceptionally strong evidence from unbiased observational studies	1.1.1.1.1.1.1.1.18. Alternative approaches likely to be better for some patients under some circumstances. Further research (if performed) is likely to have an important impact on our confidence in the estimate of effect and may change the estimate
**Low-quality evidence, weak recommendation**	1.1.1.1.1.1.1.1.19. Uncertainty in the estimates of desirable effects, harms, and burden; desirable effects, harms, and burden may be closely balanced	1.1.1.1.1.1.1.1.20. Evidence for at least one critical outcome from observational studies, RCTs with serious flaws or indirect evidence	1.1.1.1.1.1.1.1.21. Other alternatives may be equally reasonable. Further research is very likely to have an important impact on our confidence in the estimate of effect an is likely to change the estimate
**Very low-quality evidence, weak recommendation**	1.1.1.1.1.1.1.1.22. Major uncertainty in the estimates of desirable effects, harms, and burden; desirable effects may or may not be balanced with undesirable effects	1.1.1.1.1.1.1.1.23. Evidence for at least one critical outcome from unsystematic clinical observations or very indirect evidence	1.1.1.1.1.1.1.1.24. Other alternatives may be equally reasonable. Any estimate of effect, for at least one critical outcome, is very uncertain

### Agreement on statements and recommendations

A modified Delphi methodology was implemented to reach an agreement among the experts on all statements and recommendations [20]. Each was subject to voting by the experts’ panel using the Google Forms online platform. When unanimous consensus was not reached, supporting evidence from the systematic review of the literature performed for the specific research question was presented and discussed, and, if necessary, a second round of voting was carried out. The statements and recommendations were approved only if ≥ 70% expert agreement was achieved (Table 2).

**Table 2:** Summary of the 2021 SICG-SIFIPAC-SICE-WSES multidisciplinary consensus on the management of rectal cancer in the elderly. Statements and recommendations

**Consensus Topic: A. Epidemiology**	
**Key Question: 1.** Socioeconomic burden. In elderly patients with rectal cancer, how does pre-existing frailty affect the incidence of adverse events and healthcare costs?	
**Statement:** Frailty should not be considered a contraindication to surgery in elderly patients with rectal cancer. It is instead a condition that requires a correct choice of the proper surgical technique and a careful peri-operative care to reduce complication rate and consequently healthcare costs.	1.1.1.1.1.1.1.1.25. **No Recommendation**
**Agreement:** 94.1%	
**Consensus Topic: A. Epidemiology**	
**Key Question: 2.** Screening strategies. In elderly patients with rectal cancer, how do the current screening strategies compared with no screening affect prognosis?	
**Statement:** The potential benefits of screening for rectal cancer in elderly patients may vary broadly with age, life expectancy, and screening modalities. Life expectancy and comorbidity should be carefully considered in this context. Subject testing negative for screening, especially after negative colonoscopy, could consider discontinuing screening tests at the age of 75 years.	1.1.1.1.1.1.1.1.26. **Recommendation:** The experts’ panel recommends against screening in patients older than 85 years. We suggest a careful selection on an individual basis for patients between the ages of 76 and 85 years, according to their health status **(*Strong recommendation, Moderate quality of evidence—1B*).**
**Agreement:** 88.2%	
**Consensus Topic: B. Pre-intervention strategies**	
**Key Question: 3.** Improvement strategies for patient involvement in healthcare decision-making. In elderly patients with rectal cancer, how do the strategies for patient involvement in healthcare decision-making compared with the standard decision-making pathways affect compliance to planned treatments?	
**Statement:** In elderly patients with rectal cancer, the will of the patient to be involved in the decision-making process should be investigated to improve patients’ adherence to planned treatments.	1.1.1.1.1.1.1.1.27. **Recommendation:** The experts’ panel recommends the adoption of strategies for patient involvement in healthcare decision-making, the evaluation of the social background, and a discussion with the patient about therapeutic modalities for rectal cancer **(*Strong recommendation, Moderate quality of evidence—1B*).**
**Agreement:** 94.1%	
**Consensus Topic: B. Pre-intervention strategies**	
**Key Question: 4.** Frailty assessment and multidisciplinary evaluation. In elderly patients with rectal cancer, how does the frailty assessment compared with standard assessment strategies influence the outcomes of neoadjuvant treatment, surgical care, recovery, and oncological outcomes?	
**Statement:** No study directly compared the outcomes of rectal cancer treatment after frailty vs. standard assessment in patients aged above 70 years; however, despite its limitations, the literature shows that frailty, but not age, is an independent risk factor for mortality, morbidity, and re-admissions after rectal cancer surgery, radiotherapy, and palliative chemotherapy for metastatic disease.	1.1.1.1.1.1.1.1.28. **Recommendation:** The expert’s panel suggests the use of a frailty score in the preoperative evaluation of rectal cancer patients above 70 years of age **(*Weak recommendation, Low quality of evidence—2C*)*.***
**Agreement:** 97.1%	
**Consensus Topic: B. Pre-intervention strategies**	
**Key Question: 5.** Definition and prioritization of patient-centered outcomes. In elderly patients with rectal cancer, how does the prioritization of patient-centered outcomes compared to standard reported outcomes influence the treatment strategies?	
**Statement:** When deciding the best therapy for elderly patients with rectal cancer, many factors should be considered, such as preoperative frailty and functional status, operative curability, tumor stage, co-morbidities, life expectancy, and patient desire.	1.1.1.1.1.1.1.1.29. **Recommendation:** The experts’ panel recommends involving elderly patients with rectal cancer in a shared decision-making process for the therapeutic pathway with a “two-way communication” between healthcare-professionals and patients/caregivers **(*Strong recommendation, Moderate quality of evidence—1B*).**
**Agreement:** 97.1%	
**Consensus Topic: C. Diagnosis and staging**	
**Key Question: 6.** In elderly patients with rectal cancer, how does pelvic Magnetic Resonance Imaging (MRI) perform, compared to Endoscopic Ultrasonography (EUS), in the staging and re-staging following neoadjuvant therapy?	
**Statement:** Both EUS and MRI provide reasonable diagnostic accuracy in the staging of rectal cancer. However, EUS outperforms MRI in overall T, overall N, T1 and T3 staging. Morphological re-assessment of T- or N-stage by MRI or EUS after neoadjuvant therapy is currently not accurate or consistent enough for clinical application. EUS is slightly superior to MRI in re-staging the T category after neoadjuvant therapy, whereas the re-assessment by MRI before surgery appears to have a clinical role in excluding circumferential resection margin involvement. The multidisciplinary team should be aware of advantages and disadvantages of both modalities and choose the appropriate method while considering diagnostic accuracy of each test in each specific condition.	**Recommendation:** The experts’s panel recommends that either EUS or MRI should be used based on local availability and expertise. MRI has relatively high diagnostic accuracy for preoperative circumferential resection margin assessment and should be used for accurate preoperative staging when muscularis propria invasion and adjacent organ invasion is suspected. Given the operating characteristics of EUS and MRI and lack of consensus in guidelines, clinical decision may ultimately be determined by access to resources, local expertise, and institutional policy **(*Strong recommendation, Moderate quality of evidence—1B*).**
**Agreement:** 85.3%	
**Consensus Topic: D. Neoadjuvant therapy**	
**Key Question: 7.** Indication, timing, compliance, and outcomes of neoadjuvant therapy. In elderly patients with locally advanced stage II-III resectable rectal cancer, how does short-course radiotherapy compared to standard neoadjuvant chemo-radiotherapy affect the oncological outcome?	
**Statement:** Preoperative short-course radiotherapy (PSCRT) and preoperative long-course chemo-radiotherapy (PLCCRT) are both effective as neoadjuvant treatments for locally advanced stage II-III resectable rectal cancer. The primary advantage of PSCRT is its lower toxicity compared with PLCCRT. This advantage could be particularly relevant in frail elderly patients with rectal cancer. PSCRT with delayed (more than 4 weeks) surgery may be an effective strategy for elderly and frail patients with locally advanced stage II-III resectable rectal cancer who have a poor performance status or significant comorbidities.	1.1.1.1.1.1.1.1.30. **Recommendation:** The experts’ panel suggests short-course radiotherapy with delayed surgery for more than 4 weeks in elderly frail patients with locally advanced stage II-III resectable rectal cancer **(*Weak recommendation, Moderate quality of evidence—2B*).**
**Agreement:** 87.9%	
**Consensus Topic: E. Surgery**	
**Key Question: 8.** Prehabilitation, Enhanced Recovery After Surgery (ERAS). In elderly patients with rectal cancer, how do ERAS pathways compared to standard practice affect early surgical outcomes and recovery?	
**Statement:** As the ERAS protocol is conceived to improve postoperative outcomes independently from age, it is intuitive to conclude that older patients could benefit from the correct application of ERAS protocols. The importance of assessing frailty in surgical patients appears to be of crucial importance to assure the correct implementation and adherence to the protocols.	1.1.1.1.1.1.1.1.31. **Recommendation:** The experts’ panel suggests that ERAS protocols should be always implemented for elderly patients undergoing rectal surgery, regardless of age. A correct evaluation of frailty should be performed before surgery in order to obtain the maximum benefit from the application of the protocol in elderly population **(*Weak recommendation, Moderate quality of evidence—2B*).**
**Agreement:** 97.1%	
**Consensus Topic: E. Surgery**	
**Key Question: 9.** Oral antibiotic prophylaxis. In elderly patients with rectal cancer, how does oral plus intravenous antibiotic prophylaxis affect the rate of surgical site infection (SSI) compared to intravenous antibiotic prophylaxis only?	
**Statement:** Current evidence suggests a potentially significant role for oral antibiotic prophylaxis, either in combination with mechanical bowel preparation or alone, in the prevention of postoperative complications in elective colorectal surgery. In elderly patients, oral plus intravenous antibiotic prophylaxis may improve the rate of surgical site infection.	1.1.1.1.1.1.1.1.32. **Recommendation:** The experts’ panel recommends that in elderly patients with rectal cancer, oral plus intravenous antibiotic prophylaxis should be preferred over intravenous antibiotic prophylaxis alone in order to reduce postoperative SSIs **(*Strong recommendation, Moderate quality of evidence—1B*).**
**Agreement:** 94.1%	
**Consensus Topic: E. Surgery**	
**Key Question: 10.** Local excision. In elderly patients with T1 low rectal cancer, how does local excision with curative intent affect functional and oncological outcomes compared to rectal resection?	
**Statement:** In elderly patients with T1 low rectal cancer, local excision with curative intent does not affect long-term functional outcomes. Patients aged > 70 do not show consistent variations of anorectal function after the excision of T1 low rectal cancer without neoadjuvant radiotherapy. Full thickness local excision of T1 rectal cancer can be applied safely in elderly patients with oncological results that are comparable to radical surgery if the pre-operative selection is accurate. If high risk features are present, the choice of local excision has to be made on a case by case basis and balanced with the operative risk. The possibility to administer adjuvant therapy in this case should be considered.	1.1.1.1.1.1.1.1.33. **Recommendation:** The experts’ panel suggests to consider local excision as a valid alternative to Total Mesorectal Excision (TME) among the therapeutic options for T1 rectal cancer in elderly frail patients, due to promising functional and oncological outcomes **(*Weak recommendation, Moderate quality of evidence—2B*).**
**Agreement:** 97.1%	
**Consensus Topic: E. Surgery**	
**Key Question: 11.** In elderly patients with a low invasive rectal cancer, how does local excision with palliative intent, if feasible, affect functional and oncological outcomes compared to rectal resection with TME?	
**Statement:** Local excision is used in combination with neoadjuvant chemo-radiotherapy as an alternative tool to major resection in more advanced rectal cancer. Even if a study specifically addressing the elderly population does not currently exist, the mean age of patients undergoing such a management is higher than those receiving TME. In this case, anorectal function after excision may be affected by the radiation therapy but still seems to be better than in TME patients. Regarding oncological outcomes, there seems to be no difference between radical TME and local excision with palliative purposes.	1.1.1.1.1.1.1.1.34. **Recommendation:** The experts’ panel suggests to consider local excision as a palliative approach in elderly patients when they are judged unfit for major surgery, in combination with neoadjuvant therapy, when feasible **(*Weak recommendation, Low quality of evidence—2C*).**
**Agreement:** 91.2%	
**Consensus Topic: E. Surgery**	
**Key Question: 12.** Local Excision. In elderly patients with a cT2/T3 N0 low rectal cancer, how does radiotherapy followed by local excision compared to rectal resection with TME affect functional and oncological outcomes?	
**Statement:** In elderly patients with a small cT2/T3 N0 low rectal cancer, radiotherapy followed by local excision in clinically good responders may offer no long term difference in oncological outcomes compared to TME. In elderly patients with a cT2/T3 N0 low rectal cancer, radiotherapy followed by local excision may offer impaired functional outcomes, but in any case better than after TME.	1.1.1.1.1.1.1.1.35. **Recommendation:** The panel recommends to consider elderly patients with small cT2/T3 N0 low rectal cancers suitable for neoadjuvant therapy and organ sparing transanal local excision following chemo-radiotherapy **(*Strong recommendation, Moderate quality of evidence—1B*).**
**Agreement:** 91.2%	
**Consensus Topic: E. Surgery**	
**Key Question: 13.** Local Excision. In elderly patients who underwent local excision of a sessile polyp of the low rectum, with an unexpected result of a pT2/T3 Nx cancer on the resultant histopathology, how does postoperative radiotherapy compare to rectal resection with TME in terms of functional and oncological outcomes?	
**Statement:** In elderly fit patients who underwent local excision for a low rectal sessile polyp with final pathology of pT2/T3 rectal cancer, radical surgery with TME is the treatment of choice. However, in case of contraindication to major surgery due to comorbidities, other treatments should be considered including adjuvant radiotherapy. The accurate definition of the surgical risk is a key point to guide towards the most appropriate decision.	1.1.1.1.1.1.1.1.36. **Recommendation:** The experts’ panel recommends radical surgery with TME as treatment of choice in elderly patients fit for surgery after the local excision of a sessile polyp of the low rectum subsequently confirmed as a pT2/T3 Nx cancer on the histopathology result **(*Strong recommendation, Moderate quality of evidence—1B*).**
**Agreement:** 96.8%	
**Consensus Topic: E. Surgery**	
**Key Question: 14.** Minimally invasive surgery (laparoscopic/robotic TME, TaTME). In elderly patients with rectal cancer, how does minimally invasive surgery (laparoscopic/robotic-assisted) compared to open surgery affect recovery, functional and oncological outcomes?	
**Statement:** In elderly fit patients with rectal cancer, a consistent amount of evidence suggests that laparoscopic TME is safe and feasible and is associated with short-term benefits compared to open surgery. There is insufficient evidence to support potential benefits of robotic and transanal approaches for rectal cancer resection in elderly patients compared to laparoscopy or open surgery.	1.1.1.1.1.1.1.1.37. **Recommendation:** The experts’ panel suggests laparoscopic TME in elderly fit patients with rectal cancer after a careful evaluation of patient’s medical history, performance status, and tumor characteristics **(*Weak recommendation, Moderate quality of evidence—2B*).** Minimally invasive surgery approaches other than laparoscopy and open surgery may be considered for TME in elderly patients with rectal cancer after a careful evaluation of patient’s medical history, performance status, and tumor characteristics. Open surgery may be appropriate in selected cases, including locally advanced tumors, multiple previous abdominal operations, or previous pelvic surgery. **(*Neutral recommendation due to very limited and low-quality evidence*).**
**Agreement:** 96.8%	
**Consensus Topic: E. Surgery**	
**Key Question: 15.** Early versus delayed ileostomy closure. In elderly patients with low rectal cancer who underwent low anterior resection with diverting loop ileostomy, how does early ileostomy closure compared to delayed ileostomy closure affect complications and quality of life?	
**Statement:** In elderly patients with low rectal cancer who underwent low anterior resection with diverting loop ileostomy, early ileostomy closure is safe and feasible. Early closure is related with lower incidence of postoperative small bowel obstruction, stoma-related complications and better functional outcomes, despite a relatively higher surgical site infection rate compared with late closure.	1.1.1.1.1.1.1.1.38. **Recommendation:** The experts’ panel suggests that in selected elderly fit patients, early (within 2 weeks) closure of ileostomy after rectal resection should be performed. **(*Weak recommendation, Moderate quality of evidence—2B*).**
**Agreement:** 87.9%	
**Consensus Topic: F. Watch and wait**	
**Key Question: 16.** Watch and wait, indications and outcomes. In elderly patients with rectal cancer, how does the watch and wait strategy in case of absence of clinically detectable residual tumor after neoadjuvant therapy affect functional and oncological outcomes compared to rectal resection?	
**Statement:** In elderly patients with rectal cancer, in case of complete clinical response after neoadjuvant therapy, watch and wait may be considered a safe strategy, especially in selected patients, such as frail patients and patients with low-rectal tumors, with comparable oncological outcomes and better functional results in comparison to surgery.	1.1.1.1.1.1.1.1.39. **Recommendation:** The experts’ panel suggests a watch and wait strategy in selected frail elderly patients with low-rectal tumors in case of complete clinical response after neoadjuvant therapy. A stringent surveillance protocol, at least in the first 3 years, and a candid discussion with the patient about the potential risks of this strategy are recommended **(*Weak recommendation, Low quality of evidence—2C*).**
**Agreement:** 97.0%	
**Consensus Topic: G. Adjuvant chemotherapy**	
**Key Question: 17.** Adjuvant chemotherapy. In elderly patients with rectal cancer who underwent radical surgery with curative intent, does fluoropyrimidine-based adjuvant chemotherapy improve the oncological outcome compared with clinical and radiological follow-up?	
**Statement:** There is little evidence to support benefit of adjuvant chemotherapy for elderly patients with rectal cancer who have undergone radical surgery with curative intent compared with clinical and radiological follow-up.	**Recommendation:** The experts’ panel suggests that for selected stage III and stage II high-risk elderly patients with rectal cancer who underwent radical surgery with curative intent, a fluoropyrimidine-based adjuvant chemotherapy should be preferred to clinical and radiological follow-up. Decision to perform adjuvant chemotherapy (alone or associated with radiotherapy) has to be taken after a multidimensional and geriatric assessment and must be shared within the multidisciplinary board, taking into account individual cancer risk of recurrence, DYPD evaluation, previous treatment (surgery alone or preoperative chemo-radiotherapy), patient’s performance status and comorbidities **(*Weak recommendation, Low quality of evidence—2C*).**
**Agreement:** 93.8%	
**Consensus Topic: H. Liver disease**	
**Key Question: 18.** Treatment of synchronous liver metastases: In elderly patients with rectal cancer, how do sequential resections (liver then rectum, or vice-versa) compared to simultaneous resection affect postoperative morbidity, mortality, and oncological outcomes?	
**Statement:** Liver resections in elderly patients aged > 75 years with colorectal liver metastases show equivalent disease-free survival compared with younger patients, although in these patients perioperative mortality is almost doubled and overall morbidity rate seems to be higher. Simultaneous and staged colorectal and hepatic resections for synchronous liver metastases have comparable postoperative morbidity and mortality, recurrence rate, and 5-year overall survival. However, the simultaneous approach seems to be safe only in selected elderly patients with less severe liver disease. Patients with a high burden of liver disease may be more likely to benefit from early liver-first approach after down-staging therapy.	1.1.1.1.1.1.1.1.40. **Recommendation:** The experts’ panel suggests staged or simultaneous liver resection for colorectal liver metastases in elderly patients depending on the burden of liver disease and patient’s frailty status. Caution should be taken in performing major hepatectomies in patients aged > 75 years, given the increase in postoperative morbidity and mortality **(*Weak recommendation, Moderate quality of evidence—2B*).**
**Agreement:** 97.1%	
**Consensus Topic: I. Emergency presentations**	
**Key Question: 19.** Obstructing rectal cancer. In elderly patients with obstructing upper rectal cancer, how does bridge-to-surgery rectal stent placement compared to emergency surgery affect oncological outcomes and the rate of minimal access surgery?	
**Statement:** In elderly patients with obstructing upper rectal cancer, bridge-to-surgery rectal stent placement (when possible) compared to emergency surgery could improve short-term results, even potentially increasing the rate of minimal access surgery, with similar disease-free and overall survival rates.	1.1.1.1.1.1.1.1.41. **Recommendation:** The experts’ panel suggests that in elderly patients with obstructing upper rectal cancer, bridge-to-surgery rectal stent placement (when possible) should be preferred over emergency surgery **(*Weak recommendation, Moderate quality of evidence—2B*).**
**Agreement:** 82.4%	

## Results

### Consensus Topic: A. Epidemiology

**Key Question 1.** Socioeconomic burden. In elderly patients with rectal cancer, how does pre-existing frailty affect the incidence of adverse events and healthcare costs?

**Statement.** Frailty should not be considered a contraindication to surgery in elderly patients with rectal cancer. It is instead a condition that requires a correct choice of the proper surgical technique and a careful peri-operative care to reduce complication rate and consequently healthcare costs.

**Recommendation.** No Recommendation.

**Agreement:** 94.1%

About 60% of colorectal cancers develop in patients over 65 years old [21, 22]. Around 25–45% of these patients can be considered frail [22–24]. Such a wide variability is due to the lack of consensus on the definition of “frailty.” Results of several studies regarding the postoperative outcome of frail patients showed that:

*A.* There is a higher risk of medical and surgical postoperative complications, especially severe ones, in this subgroup of patients than in the general population [21–28]. The incidence of these complications ranges from 6% [29, 30] to over 65% [29, 31].

*B.* There is a higher risk of sepsis in frail patients [21, 27].

*C.* Frail elderly patients who underwent colorectal resection for cancer have a more prolonged hospital stay [21, 22], which ranges from 5 days [29, 32] to more than 20 days [29, 33].

*D.* There is higher postoperative mortality in elderly frail patients, especially in the first 30 days after the surgical intervention [21, 27, 28].

*E.* Elderly patients with colorectal cancer who underwent surgery have a higher hospital readmission rate within 30 days [21, 25].

Strong evidence emphasizes that postoperative complications lead to higher healthcare costs. The increment ranges from 1.500 USD to 18.000 USD [29, 33]. Length of hospital stay, obviously influenced by complications, is the first condition that causes an increment of costs [29, 30, 33–36]. There is less correlation between healthcare costs and the different surgical techniques [29]. Currently, a standardized approach for reporting costs associated with complications is lacking. Moreover, there are no results regarding the costs of surgery only in elderly patients. We can suppose that healthcare costs are higher in elderly patients, assuming that most of them are frail, and for this reason, they have a higher rate of postoperative complications. A standard definition of “frailty” is needed to adopt a better surgical approach for colorectal cancer in this group of patients.

### Consensus Topic: A. Epidemiology

**Key Question 2.** Screening strategies. In elderly patients with rectal cancer, how do the current screening strategies compared with no screening affect prognosis?

**Statement.** The potential benefits of screening for rectal cancer in elderly patients may vary broadly with age, life expectancy, and screening modalities. Life expectancy and comorbidity should be carefully considered in this context. Subject testing negative for screening, especially after negative colonoscopy, could consider discontinuing screening tests at the age of 75 years.

**Recommendation.** The experts’ panel recommends against screening in patients older than 85 years. We suggest a careful selection on an individual basis for patients between the ages of 76 and 85 years, according to their health status (*Strong recommendation, Moderate quality of evidence—1B*).

**Agreement:** 88.2%

Colorectal cancer screening is recommended for average-risk individuals between the ages of 50 and 75 years. Once patients are older than 75 years, the risk-to-benefit ratio of ongoing screening begins to shift. The potential benefits of screening for rectal cancer in elderly patients may vary broadly with age, life expectancy, and screening modalities (i.e., stool-based, radiological, blood testing, and endoscopic screening). Current guidelines from the U.S. Preventive Services Task Force (USPSTF), and the American Cancer Society (ACS), recommend against screening patients for rectal cancer between the ages of 76 and 85. However, in this age group, screening could be suggested on an individual patient basis after personalized assessment. The USPSTF and the ACS also recommend against screening for individuals older than 85 years [37, 38]. Both the American Gastroenterology Association (AGA) and the American Society of Gastrointestinal Endoscopy (ASGE) guidelines reported the potential benefits of screening in patients up to 86 years if they had not been screened before. The cost-effectiveness analysis performed by van Hees et al. to assess whether screening should be considered in unscreened elderly subjects aged 76 to 90 years found that screening was cost-effective up to age 86 years. Screening with colonoscopy was indicated up to age 83 years, sigmoidoscopy was indicated at age 84 years, and the fecal immunochemical test was indicated at ages 85 and 86. Nevertheless, comorbidity should be carefully considered in this context. In unscreened individuals with severe comorbid conditions, screening was cost-effective up to age 80 years (colonoscopy indicated up to age 77 years, sigmoidoscopy at age 78 years, and fecal immunochemical test at ages 79 and 80) [39].

Subjects who tested negative for screening, especially after negative colonoscopy, could consider discontinuing screening tests at 75 years [40, 41]. A cross-sectional study showed that colonoscopy in subjects older than 80 years offered only 15% of extension in life expectancy compared to younger individuals. Therefore, it has been suggested that screening colonoscopy in very elderly patients should be carried out only after careful evaluation of risks, benefits, and patient preferences [42]. A calculation model proposed by Inadomi and Sonnenberg suggested that screening and continuous surveillance should be carried out only on subjects who have a life expectancy of 10 years or more. This model showed a more significant reduction in longevity due to rectal cancer in younger patients compared to older age groups, thereby reflecting the influence of competing risks of death from other causes that increase with age [43]. Consensus guidelines from the ASGE recommended assessments of cognition and capacity in older adults to guarantee that patients are adequately able to engage in shared decision-making [40].

### Consensus Topic: B. Pre-intervention strategies

**Key Question 3.** Improvement strategies for patient involvement in healthcare decision-making. In elderly patients with rectal cancer, how do the strategies for patient involvement in healthcare decision-making compared with the standard decision-making pathways affect compliance to planned treatments?

**Statement.** In elderly patients with rectal cancer, the will of the patient to be involved in the decision-making process should be investigated to improve patients’ adherence to planned treatments.

**Recommendation.** The experts’ panel recommends the adoption of strategies for patient involvement in healthcare decision-making, the evaluation of the social background, and a discussion with the patient about therapeutic modalities for rectal cancer (*Strong recommendation, Moderate quality of evidence—1B*).

**Agreement:** 94.1%

The standard decision-making pathway for rectal cancer management is based on international guidelines’ recommendations. However, the possibility to adhere to standard therapeutic schemes is not systematic for elderly patients due to higher risk for adverse events, complications, comorbidities, treatment-related mortality, and also due to explicit refusal of the patient to proceed or continue therapies, compared to younger patients [44–46]. European studies demonstrate that the proportion of patients with colorectal cancer treated following national guidelines varies between 53 and 90%, with patient preference (27%) and functional status (20%) the most commonly reported reasons for adjusted treatment [46]. Nonetheless, patient involvement in perceiving personal preferences about the treatment is not systematic, especially in older patients [47]. Diefenhardt et al. showed that in patients with rectal cancer, adherence to neoadjuvant chemo-radiotherapy was significantly associated with disease-free survival [44]. Mari et al. found that adjuvant chemotherapy for locally advanced rectal cancer was associated with improved overall survival, although RCTs showed a 43 to 73% compliance rate, which may affect efficacy [45]. Adherence is influenced by treatment-related and by patient-related factors such as cognitive status and socioeconomic status. The decision-making process related to treatment should consider patients’ preferences after receiving appropriate information about risks and benefits. In elderly patients, therapeutic decisions should always be preceded by a comprehensive geriatric assessment, an evaluation of the social background and social support of the patient, a discussion with the patient about therapeutic modalities, probability of having treatment-related toxicity, and side effects [48].

However, not all patients desire to be involved in the healthcare decision-making process. Elkin et al. reported that about half of the patients desire to decide their therapeutic pathway. Besides, physicians do not always correctly perceive the patient’s will to be involved [49]. Elderly patients affected by rectal cancer should be carefully evaluated from an oncological point of view, but also the cognitive and social background should be considered. The patient’s will to be involved in therapeutic decisions should be investigated. If positive, a shared decision-making process should be structured by integrating patients’ and clinicians’ values and beliefs to recognize the “best” outcome for each specific scenario, ultimately improving patients’ outcomes [50].

### Consensus Topic: B. Pre-intervention strategies

**Key Question 4.** Frailty assessment and multidisciplinary evaluation. In elderly patients with rectal cancer, how does the frailty assessment compared with standard assessment strategies influence the outcomes of neoadjuvant treatment, surgical care, recovery, and oncological outcomes?

**Statement.** No study directly compared the outcomes of rectal cancer treatment after frailty vs. standard assessment in patients aged above 70 years; however, despite its limitations, the

literature shows that frailty, but not age, is an independent risk factor for mortality, morbidity, and re-admissions after rectal cancer surgery, radiotherapy, and palliative chemotherapy for metastatic disease.

**Recommendation.** The expert’s panel suggests the use of a frailty score in the preoperative evaluation of rectal cancer patients above 70 years of age (*Weak recommendation, Low quality of evidence—2C*).

**Agreement:** 97.1%

No study directly compared the outcomes of rectal cancer treatment after frailty vs. standard assessment in elderly patients. In older general, cardiovascular and orthopedic surgical patients, frailty predicts postoperative mortality, complications, and prolonged length of hospital stay [51]. A few studies on colorectal surgery confirmed the prognostic value of several frailty scores, but neither distinguished between colon and rectal surgery nor examined their use as a decision-making tool [26, 28, 52, 53].

The systematic review and meta-analysis by Boakye et al. included 37 cohort studies, of which 35 were on comorbidity and two on frailty. Compared to colorectal cancer patients without comorbidity, those with mild/moderate and severe comorbidity showed a higher risk of 30-day (OR = 1.71 and OR = 2.62), overall (HR = 1.41 and HR = 2.03), and cancer-specific mortality (HR = 1.06 and HR = 1.14). Similarly, higher overall mortality was reported in frail colorectal cancer patients than non-frail patients [54]. A geriatric frailty assessment can also predict 1-year and 5-year survival in older patients after colorectal surgery for cancer. In the study by Ommundsen et al., a pre-operative geriatric assessment was performed on a cohort of 178 colorectal cancer patients aged 70 and older who underwent elective surgery. The geriatric assessment resulted in patients being divided into two groups: frail or non-frail. One-year survival was 80% in the frail group and 92% in the non-frail group. Five-year survival was significantly lower in frail (24%) than non-frail patients (66%), and this difference was apparent both within the stratification on TNM stages 0–II and TNM stage III [55].

However, observational studies on rectal cancer focused on the prognostic value of frailty assessment without examining its use to tailor the management plan. They found that frailty, but not age, is an independent risk factor for mortality and morbidity [56–58] and re-admissions after rectal cancer surgery [59], radiotherapy [60], and palliative chemotherapy for metastatic disease [61].

All the referenced studies also included patients younger than 80; this is an additional limitation to the available evidence. The large number of available frailty scores and the diversity of inclusion criteria seriously limit the possibility to compare studies.

The scarce, heterogeneous literature does not allow to propose a firm statement about the key question, but a more extensive use of a frailty assessment could be advisable.

The randomized phase II GERICO trial (NCT02748811) has recently been completed. This was designed to investigate whether geriatric assessment and intervention before and during treatment with chemotherapy in frail elderly patients with stages II-IV colorectal cancer patients would increase the number of patients completing chemotherapy. The findings from the GERICO trial are expected to provide valuable knowledge about whether it is beneficial for the elderly patient undergoing chemotherapy to be treated simultaneously by a geriatric specialist [62].

### Consensus Topic: B. Pre-intervention strategies

**Key Question 5.** Definition and prioritization of patient-centered outcomes. In elderly patients with rectal cancer, how does the prioritization of patient-centered outcomes compared to standard reported outcomes influence the treatment strategies?

**Statement.** When deciding the best therapy for elderly patients with rectal cancer, many factors should be considered, such as preoperative frailty and functional status, operative curability, tumor stage, co-morbidities, life expectancy, and patient desire.

**Recommendation.** The experts’ panel recommends involving elderly patients with rectal cancer in a shared decision-making process for the therapeutic pathway with a “two-way communication” between healthcare professionals and patients/caregivers (*Strong recommendation, Moderate quality of evidence—1B*).

**Agreement:** 97.1%

Even though rectal cancer is predominantly a disease of the elderly, the treatment is not straightforward nor standardized. When deciding the best therapy for an elderly patient with rectal cancer, many factors should be considered, including the preoperative frailty and functional status, operative curability, tumor stage, comorbidities, life expectancy, and patient desire: these must all be evaluated before recommending any therapy [15, 63]. Nowadays, the lack of standardized measurement hampers the widespread attainment of value-based care for rectal cancer patients.

Treatment strategy should focus mainly on patients’ functional recovery and quality of life, rather than mere 5-year disease-free survival while maintaining appropriate oncological standards and minimizing adverse effects [64].

PROMs (patient-reported outcome measures) are crucial to be considered in real life. It is essential to choose a treatment or a combination of treatments that ensure excellent tumor control with minimal acute and late side effects to provide a personalized healthcare path and the best possible quality of life [15]. Patients should be actively involved in the decision-making with a “two-way communication” between healthcare professionals and patients/caregivers [65].

The International Consortium for Health Outcomes Measurement (ICHOM) working group has developed a consensus on the use of well-validated outcome measures, including PROMs [66]. A list of 40 outcomes was evaluated by the ICHOM working group and underwent voting. The final recommendation included survival and disease control outcomes, the disutility of care, degree of health, and quality of death. Moreover, selected case-mix factors were recommended to be collected at baseline to facilitate the comparison of results across treatments. Although age, taken as an independent variable, is not a contraindication to any specific therapy, including radical surgery [67], elderly patients with rectal cancer may present psychological disorders and have a higher incidence of poor fecal continence following surgery. The surgical intervention should be based on an accurate balance between life-expectancy and comorbid conditions, performing a careful evaluation of the cardiovascular, pulmonary, renal, metabolic, and nutritional status.

### Consensus Topic: C. Diagnosis and staging

**Key Question 6.** In elderly patients with rectal cancer, how does pelvic Magnetic Resonance Imaging (MRI) perform, compared to Endoscopic Ultrasonography (EUS), in the staging and re-staging following neoadjuvant therapy?

**Statement.** Both EUS and MRI provide reasonable diagnostic accuracy in the staging of rectal cancer. However, EUS outperforms MRI in overall T, overall N, T1 and T3 staging. Morphological re-assessment of T- or N-stage by MRI or EUS after neoadjuvant therapy is currently not accurate or consistent enough for clinical application. EUS is slightly superior to MRI in re-staging the T category after neoadjuvant therapy, whereas the re-assessment by MRI before surgery appears to have a clinical role in excluding circumferential resection margin involvement. The multidisciplinary team should be aware of advantages and disadvantages of both modalities and choose the appropriate method while considering diagnostic accuracy of each test in each specific condition.

**Recommendation.** The experts’ panel recommends that either EUS or MRI should be used based on local availability and expertise. MRI has relatively high diagnostic accuracy for preoperative circumferential resection margin assessment and should be used for accurate preoperative staging when muscularis propria invasion and adjacent organ invasion are suspected. Given the operating characteristics of EUS and MRI and lack of consensus in guidelines, clinical decision may ultimately be determined by access to resources, local expertise, and institutional policy (*Strong recommendation, Moderate quality of evidence—1B*).

**Agreement:** 85.3%

Endoscopic Ultrasonography (EUS) and Magnetic Resonance Imaging (MRI) are used for locoregional staging of rectal cancer. There is a lack of consensus on the best modality of locoregional staging, especially for small tumors, with different studies supporting both EUS and MRI. The American Society for Gastrointestinal Endoscopy (ASGE) recommends EUS for the locoregional staging of rectal cancer to guide therapy [68]. The European Society of Medical Oncology (ESMO) suggests using EUS or MRI in early T staging and suggests MRI as the optimal modality of N staging [69]. The National Comprehensive Cancer Network (NCCN) lists both MRI and EUS for clinical staging, although MRI is preferred [70].

According to some authors, MRI is the imaging of choice for the staging of locally advanced rectal cancer [71, 72] because it allows to select patients, guide the surgeon in surgical planning or for a “wait and see” approach, identify negative prognostic factors [73] such as mesorectum invasion (T3), lymph node involvement (N +), mesorectal fascia involvement and macroscopic perivascular infiltration [74].

Al-Sukhni et al. [75] and Zhang et al. [76] agree on the high specificity of MRI (93%) for identifying the infiltration of the perirectal fascia, the excellent accuracy of the method in the staging of T (T1–T2 vs. T3), differentiating the T3 initial (≤ 5-mm extra-parietal infiltration) from T3 (> 5-mm extra-parietal infiltration) with an accuracy of 91% and 88% respectively and, finally, in the high specificity of MRI (97%) in highlighting infiltration of adjacent organs and structures, defining the stage T4.

Chan et al. [77] performed a meta-analysis to compare, in the same patient population, the diagnostic accuracy, sensitivity, and specificity of EUS and MRI in the staging of rectal cancer. The pooled analysis included six studies with 234 patients. Pooled sensitivity and specificity in T staging were 0.79 and 0.89 for EUS and 0.79 and 0.85 for MRI. Pooled sensitivity and specificity in N staging were 0.81 and 0.88 for EUS and 0.83 and 0.90 for MRI, respectively. EUS outperformed MRI in overall T, overall N, T1, and T3 staging in the area under the curve analysis. Conversely, MRI was superior to EUS in T2 staging. The accuracy of EUS in detecting early-stage rectal cancer can have clinical applicability because a T1 rectal cancer can be treated by local excision.

Regarding the evaluation of local lymph node metastases, the sensitivity and specificity in patients with no neoadjuvant chemo-radiotherapy were 0.77 and 0.76 for MRI, 0.57 and 0.80 for EUS, and 0.79 0.76 for CT scan in the meta-analysis by Li et al. [78]. MRI showed higher accuracy than EUS for patients who did not receive neoadjuvant therapy. High-resolution MRI showed similar diagnostic accuracy compared to EUS and CT scan. The authors suggested the use of MRI rather than EUS for lymph node evaluation after neoadjuvant therapy. The pooled sensitivity and specificity of EUS to determine T1-stage rectal cancer was 87.8% and 98.3%, respectively, in the meta-analysis by Puli et al. [79]. For the T2 stage, EUS had a pooled sensitivity and specificity of 80.5% and 95.6%. To stage T3 rectal cancers, EUS had a pooled sensitivity and specificity of 96.4% and 90.6%. In determining the T4 stage, EUS had a pooled sensitivity of 95.4% and specificity of 98.3%.

MRI and EUS also play a role in the re-staging of locally advanced rectal cancer after preoperative chemo-radiotherapy. The accuracy of re-staging imaging is different for different T stages, and it is highest for T3 tumors. However, morphological assessment of T- or N-stage by MRI or EUS is currently not accurate or consistent enough for clinical application. The diagnostic performance of MRI, EUS, and CT scan in predicting the response of locally advanced rectal cancer after neoadjuvant therapy was assessed in the meta-analysis by de Jong et al. [80]. Forty-six studies comprising more than 2.200 patients were included. The pooled accuracy in assessing tumor response after neoadjuvant therapy was 75% for MRI, 82% for EUS, and 83% for CT scan. Pooled accuracy in detecting T4 tumors with invasion to the circumferential resection margin was 88% and 94% for EUS. Pooled accuracy in predicting the presence of lymph node metastases was 72% for MRI, 72% for EUS, and 65% for CT.

In the systematic review and meta-analysis by Zhao et al. [81], EUS was superior to MRI in the re-staging T category. The sensitivity estimate for rectal cancer diagnosis (T0) by EUS was higher than the sensitivity estimate for MRI (37.0% vs. 15.3%). For T3-T4 cancers, sensitivity estimates of MRI and EUS were comparable (82.1% vs. 87.6%), whereas specificity estimates were poor (53.5% vs. 66.4%). For lymph node involvement, there was no significant difference between the sensitivity estimates for MRI and EUS (61.8% vs. 49.8%). Specificity estimates for MRI and EUS were 72.0% vs. 78.7%. For circumferential resection margin involvement, MRI sensitivity and specificity were 85.4% and 80.0%, respectively.

Sixty-three studies were included in the systematic review and meta-analysis by Memon et al. [82]. Twelve re-staging MRI studies and 18 re-staging EUS studies were eligible for meta-analysis of T-stage and N-stage and N-status re-staging accuracy. Overall, EUS T-stage re-staging accuracy (65%) was non-significantly higher than MRI T-stage accuracy (52%). Re-staging MRI was accurate at excluding circumferential resection margin involvement. Re-staging MRI and EUS were equivalent at the prediction of nodal status (72%), with over-staging and under-staging occurring in 10-15%.

### Consensus Topic: D. Neoadjuvant therapy

**Key Question 7.** Indication, timing, compliance, and outcomes of neoadjuvant therapy. In elderly patients with locally advanced stage II-III resectable rectal cancer, how does short-course radiotherapy compared to standard neoadjuvant chemo-radiotherapy affect the oncological outcome?

**Statement.** Preoperative short-course radiotherapy (PSCRT) and preoperative long-course chemo-radiotherapy (PLCCRT) are both effective as neoadjuvant treatments for locally advanced stage II-III resectable rectal cancer. The primary advantage of PSCRT is its lower toxicity compared with PLCCRT. This advantage could be particularly relevant in frail elderly patients with rectal cancer. PSCRT with delayed (more than 4 weeks) surgery may be an effective strategy for elderly and frail patients with locally advanced stage II-III resectable rectal cancer who have a poor performance status or significant comorbidities.

**Recommendation.** The experts’ panel suggests short-course radiotherapy with delayed surgery for more than 4 weeks in elderly frail patients with locally advanced stage II–III resectable rectal cancer. (*Weak recommendation, Moderate quality of evidence—2B*).

**Agreement:** 87.9%

In terms of oncological outcomes, adding preoperative chemo-radiotherapy or radiotherapy alone shows obvious advantages for local control compared to surgery alone in patients with resectable rectal cancer. Either preoperative short-course radiotherapy (PSCRT) of 25 Gy in 5 consecutive days or preoperative long-course chemo-radiotherapy with 45–50 Gy, 1.8-2 Gy/fr with concomitant 5-FU-based chemotherapy (PLCCRT) followed by radical TME is effective for local control and are regarded as the two main standards of care for patients with high-risk rectal cancer [83, 84]. Recently, an alternative strategy known as total neoadjuvant therapy (TNT), that involves administration of CRT plus neoadjuvant chemotherapy before surgery with the goal of delivering uninterrupted systemic therapy to eradicate micrometastases, has shown promising results in locally advanced rectal cancer, with superior rates of pathologic complete response (pCR) compared with standard therapy [85].

The benefit of PSCRT, as proposed by the Swedish Rectal Cancer Trial, is a lower rate of early toxicity compared to chemo-radiotherapy [86, 87].

Five systematic reviews and meta-analyses that explored the effects of PSCRT and its optimized schemes compared to PLCCRT have been published to date.

The meta-analysis by Ma et al. [88] indicated that PSCRT could be considered the treatment of choice compared to PLCCRT when a complete response is not the primary aim. PLCCRT, in fact, showed a better pCR rate (OR = 0.05, P < 0.01), although this benefit did not translate into a higher sphincter preservation rate (OR = 1.62, P = 0.25). Moreover, this meta-analysis indicated that the insufficiency of PSCRT on pCR might be improved by delayed surgery or adding consolidation chemotherapy. The two strategies had equivalent rates of post-treatment complications (OR = 1.19, P = 0.30), although patients who received PSCRT had a significantly lower incidence of total acute toxicities compared to PLCCRT (OR = 0.09, P < 0.01). In terms of long-term oncological outcomes, the two strategies showed similar tendencies of overall survival, disease-free survival, local recurrence, and distant metastases.

The systematic review and meta-analysis by Qiaoli et al. analyzed seven studies (4.973 patients) comparing PSCRT with delayed surgery (more than 4 weeks) and standard PLCCRT for locally resectable rectal cancer [89]. The pooled analysis showed that there was no statistically significant difference in overall survival (HR = 1.30, P = 0.52), disease-free survival (HR = 1.10, P = 0.64), pCR (RR = 0.74, P = 0.39), early postoperative complications (RR = 1.21, P = 0.16), treatment-related grade 3-4 toxicity (RR = 0.78, P = 0.68), local recurrence (RR = 1.27, P = 0.70) and distant metastasis (RR = 1.06, P = 0.58). However, a subgroup analysis revealed that PSCRT without adjuvant chemotherapy resulted in lower treatment-related grade 3-4 toxicity than PLCCRT (RR = 0.19, P < 0.01), but also resulted in significantly lower overall survival (HR = 2.05, P = 0.02) and pCR (RR = 1.37, P = 0.14). Regarding long-term survival, the 2018 meta-analysis by Wang et al. [90] found that there was no significant difference in overall survival (HR = 0.92, P = 0.44), disease-free survival (HR = 0.94, P = 0.50) and local recurrence (OR = 0.73, P = 0.73) between PSCRT and PLCCRT (High overall quality of evidence in the subgroup analysis of RCTs).

Twelve trials were included in the meta-analysis by Zhou et al. [91] that demonstrated no significant difference in overall survival, disease-free survival, local recurrence rate, distant metastasis rate, sphincter preservation rate, R0 resection rate, and late toxicity comparing patients who underwent PSCRT and PLCCRT. Similarly to other meta-analyses, PLCCRT increased the rate of grade 3-4 acute toxicity (RR = 0.13, P < 0.00001) and pCR (RR = 0.15, P = 0.003). Similar outcomes have been found in a recent systematic review and meta-analysis performed by the Chinese group of Yu et al. [92]. Sixteen studies with a total of 2.773 rectal cancer patients were included in the pooled analysis. There were no significant differences between PSCRT and PLCCRT concerning pCR (RR = 0.54), tumor down-staging (RR = 0.83), local recurrences (RR = 0.55), distant metastases (RR = 1.03), mortality (RR = 0.95), and serious late toxicity (RR = 1.10). However, in the subgroup analysis of RCTs, PLCCRT had a better pCR and tumor down-staging rate than PSCRT.

All the analyzed studies also included patients younger than 80. This is a limitation to the available evidence, as no study included in the pooled analyses directly compared the outcomes of PSCRT and PLCCRT for elderly patients with rectal cancer.

Recently, the preliminary results of the phase III NACRE (Neoadjuvant Treatment for Advanced Rectal Carcinoma) trial have been published. The NACRE RCT enrolled patients aged 75 and older to compare PSCRT only and standard PLCCRT. One hundred patients from 29 sites were randomized from 01/2016 to 08/2019. The median age was 80 years. The R0 resection rate in the two study arms was comparable. With a median follow-up of 15.8 months, the six-month death rate was 10.0% in the PLCCRT arm and 3.92% in the PSCRT arm. There was a significant difference in overall survival between the two arms in favor of the PSCRT arm (P = 0.04, LogRank test), and there was a trend in favor of the PSCRT arm for specific survival (P = 0.06 LogRank test). Conversely, disease-free survival was not statistically different [93].

PSCRT might be related to better health-related quality of life outcomes, according to some authors. Wiltink et al. found that patients who received a short-course scheme had a lower level of nausea/vomiting [94]. Three trials included in the meta-analysis by Ma et al. used the European Organization for Research and Treatment of Cancer (EORTC) QLQ-C30 questionnaire to evaluate the quality of life in rectal cancer patients who received PSCRT or PLCCRT. All the trials showed no statistically significant difference in the quality of life outcomes comparing the two regimens based on the scores of QLQ-C30 [94–97].

Although both the NCCN guidelines [98] and the European Society for Medical Oncology (ESMO) guidelines [69] recommend PSCRT as one of the standard treatments for locally advanced stage II-III resectable rectal cancer, neither provides an optimal time interval between the end of radiotherapy and surgery. There are two standard time intervals at which PSCRT and surgery are performed: PSCRT followed by immediate surgery within ten days, and PSCRT followed by delayed surgery (at least 4 weeks after the last radiotherapy is completed). The meta-analysis by Wu et al. [99] analyzed five studies (1.244 patients) comparing the immediate surgery (< 4 weeks) and delayed surgery (> 4 weeks) strategies as optimal interval time after PSCRT for stage II-III resectable rectal cancer patients. The delayed surgery group had a markedly higher pCR (RR = 15.71, P = 0.007), and down-staging rates (RR = 2.63, P < 0.00001), a higher proportion of patients with adjuvant pathologic stage 0 + 1 disease (RR = 1.49, P < 0.0001) and a lower incidence of postoperative complications (RR = 0.81, P = 0.008) compared with the immediate surgery group. The survival rate, sphincter preservation rate, and R0 resection rate were equivalent between the two groups.

Patients who have just undergone neoadjuvant treatment, especially the elderly and frail ones, might be in poor physical condition, and a delay in surgery may enable these patients to recover and overcome the acute radiation toxicity. A long waiting period > 4 weeks can also enable patients to improve their lifestyle, such as cease smoking, control blood pressure and diabetes, and obtain adequate nutritional support.

### Consensus Topic: E. Surgery

**Key Question 8.** Prehabilitation, Enhanced Recovery After Surgery (ERAS). In elderly patients with rectal cancer, how do ERAS pathways compared to standard practice affect early surgical outcomes and recovery?

**Statement.** As the ERAS protocol is conceived to improve postoperative outcomes independently from age, it is intuitive to conclude that older patients could benefit from the correct application of ERAS protocols. The importance of assessing frailty in surgical patients appears to be of crucial importance to assure the correct implementation and adherence to the protocols.

**Recommendation.** The experts’ panel suggests that ERAS protocols should be always implemented for elderly patients undergoing rectal surgery, regardless of age. A correct evaluation of frailty should be performed before surgery in order to obtain the maximum benefit from the application of the protocol in elderly population. (*Weak recommendation, Moderate quality of evidence—2B*).

**Agreement:** 97.1%

With an increase in life expectancy and improved quality of medical care, the number of elderly patients increases every year [100]. In a large database analysis, Jafari et al. [101] reported that 63.8% of surgical operations for colorectal cancer had been performed on patients 65 years or older and 22.6% on patients 80 years or older. Considering the increasing number of elderly patients, the safety of implementing ERAS protocols in this population has been questioned. Elderly patients may have more postoperative complications and take a longer time to recover [102–104]. However, with the implementation of ERAS protocols, patients have recovered from their operations faster, often with lower morbidity and mortality [105–107]. A systematic review of 16 studies confirmed the safety of ERAS in elderly patients who underwent colorectal surgery [108]. Before this, two RCTs found that the average length of hospital stay for elderly patients who underwent colorectal resections following an ERAS pathway was significantly lower than elderly non-ERAS patients (5.5 vs. 7 days and 9 vs. 13 days, respectively for each RCT) [109, 110].

Another retrospective study published in 2020 reviewed the outcomes of colectomy patients concerning the pre-operative assessment of frailty. The study found that with the implementation of newer modalities in the ERAS pathways, the median length of stay was three days for elderly patients and two days for non-elderly patients. The authors demonstrated that congestive heart failure increases the chances of a prolonged length of stay in elderly patients [111]. Therefore, they concluded that it is crucial to ensure that patient’s comorbidities are well controlled during hospital admission, especially among elderly patients. Furthermore, although the study showed that frail patients are discharged earlier than in other studies, the elderly patients’ progress through the ERAS pathway is still slower than in the younger cohort, often requiring prolonged hospitalization. More frail patients with many comorbidities have exhibited higher morbidity and mortality with increased hospital services utilization due to their lowered functional status [111–113]. These findings emphasize the importance of an individualized ERAS approach to elderly patients and suggest that ERAS protocols should be modified for older patients with higher frailty scores before colorectal procedures.

Moreover, elderly patients with high frailty indices require close post-discharge follow-up and communication with their primary care physicians. Another more recent systematic review was aimed to analyze the outcomes of the ERAS care pathway in older patients. The authors found how the reported adherence to the protocol items in > 65 years old patients was low to moderate, and these data could invalidate the available results. However, from the available literature emerges how ERAS strategy has significantly better outcomes than conventional care, with comparable postoperative morbidity in the younger and older patient population in the majority of the studies. This review’s most critical methodological flaw was that only six studies included older patients or subgroups of older patients. Furthermore, the older patients included may be subject to selection bias, as mainly physically and mentally fit patients tend to be recruited in the included studies [114].

### Consensus Topic: E. Surgery

**Key Question 9.** Oral antibiotic prophylaxis. In elderly patients with rectal cancer, how does oral plus intravenous antibiotic prophylaxis affect the rate of surgical site infection (SSI) compared to intravenous antibiotic prophylaxis only?

**Statement.** Current evidence suggests a potentially significant role for oral antibiotic prophylaxis, either in combination with mechanical bowel preparation or alone, in the prevention of postoperative complications in elective colorectal surgery. In elderly patients, oral plus intravenous antibiotic prophylaxis may improve the rate of surgical site infection.

**Recommendation.** The experts’ panel recommends that in elderly patients with rectal cancer, oral plus intravenous antibiotic prophylaxis should be preferred over intravenous antibiotic prophylaxis alone in order to reduce postoperative SSIs (*Strong recommendation, Moderate quality of evidence—1B*).

**Agreement:** 94.1%

Surgical site infection (SSI) is a common complication after colorectal surgery. SSI represents not only a costly expense to health services but, more importantly, influences patient recovery and survival [115]. Various strategies have been adopted in attempts to reduce postoperative SSI rates. The value of i.v. antibiotics in the immediate preoperative period is established, and they are currently used worldwide. To reduce the risk of infection after colorectal surgery, the role of oral antibiotic preparation (OAP) with or without mechanical bowel preparation (MBP) has been a matter of debate in the last ten years.

In the meta-analysis by Bellows et al., which included sixteen RCTs published between 1979 and 2007, patients randomly assigned to an oral non-absorbable antibiotic in addition to an intravenous antibiotic had a reduced risk of SSI (RR = 0.57, P = 0.0002) compared with patients receiving only intravenous antibiotics. Moreover, the use of oral non-absorbable antibiotics in addition to intravenous antibiotics had no significant effect on organ-space infections or the risk of the anastomotic leak [116].

Similarly, current evidence from the largest meta-analysis published to date on the argument suggests a potentially significant role for OAP preparation, either in combination with MBP or alone, in the prevention of postoperative complications in elective colorectal surgery. The pooled analysis was conducted on a total of 40 studies with 69.517 patients (28 randomized controlled trials and 12 cohort studies). The combination of MBP plus OAP versus MBP alone was associated with a significant reduction in SSI (RR = 0.51, P < 0.00001), anastomotic leak (RR = 0.62, P < 0.00001), 30-day mortality (RR = 0.58, P < 0.0001), overall morbidity (RR = 0.67, P < 0.00001), and postoperative ileus (RR = 0.72, P = 0.04), with no difference in *Clostridium difficile* infection rates. When a combination of MBP+OAP was compared with OAP alone, no significant difference was seen in SSI or anastomotic leak rates, but there was a significant reduction in 30-day mortality and postoperative ileus incidence with the combination [117].

Concerns regarding hospital-acquired infections (HAIs), including *Clostridium difficile*, are relevant, especially in elderly patients. However, meta-analyses [118, 119] have demonstrated the effectiveness of OAP in association with i.v. antibiotic prophylaxis with or without MBP regarding SSI risk.

In the meta-analysis by Khorasani et al., the incidence of postoperative *Clostridium difficile* infection in adults receiving oral antibiotics versus no oral antibiotics was used as the primary outcome. Fourteen RCTs and 13 cohort studies comparing bowel preparation with oral antibiotics to those without oral antibiotics were identified. The pooled OR from 4 eligible RCTs was suggestive of a greater odds of *Clostridium difficile* infection in the oral antibiotic group (OR = 4.46), with an extremely low absolute incidence of *Clostridium difficile* infection (total 11 events among 2753 patients). Conversely, the pooled OR from 6 eligible cohort studies did not demonstrate a significant difference in the odds of *Clostridium difficile* infection with, again, a very low absolute incidence of infection (total 830 events among 59.960 patients). Since the incidence of *Clostridium difficile* infection in patients who undergo colorectal surgery is very low regardless of bowel preparation regimen used, considering the beneficial role of oral antibiotics in reducing SSI, the fear for *Clostridium difficile* infection is not sufficient to omit oral antibiotics in patients undergoing colorectal resection [120].

Most studies have used the combination of an aminoglycoside (neomycin or kanamycin) with a macrolide such as erythromycin or with metronidazole. The use of such antibiotics limited to the day before surgery would reduce the risk of HAIs and antimicrobial resistance. Recently, an RCT (ORALEV) [121] about the use of OAP in the setting of elective colorectal resections was published, demonstrating that the administration of oral antibiotics as prophylaxis the day before colon surgery significantly reduces the incidence of SSI without mechanical bowel preparation and should be routinely adopted before elective colorectal surgery.

### Consensus Topic: E. Surgery

**Key Question 10.** Local excision. In elderly patients with T1 low rectal cancer, how does local excision with curative intent affect functional and oncological outcomes compared to rectal resection?

**Statement.** In elderly patients with T1 low rectal cancer, local excision with curative intent does not affect long-term functional outcomes. Patients aged > 70 do not show consistent variations of anorectal function after the excision of T1 low rectal cancer without neoadjuvant radiotherapy. Full-thickness local excision of T1 rectal cancer can be applied safely in elderly patients with oncological results that are comparable to radical surgery if the pre-operative selection is accurate. If high-risk features are present, the choice of local excision has to be made on a case by case basis and balanced with the operative risk. The possibility to administer adjuvant therapy in this case should be considered.

**Recommendation.** The experts’ panel suggests to consider local excision as a valid alternative to total mesorectal excision (TME) among the therapeutic options for T1 rectal cancer in elderly frail patients, due to promising functional and oncological outcomes (*Weak recommendation, Moderate quality of evidence—2B*).

**Agreement:** 97.1%

The current literature available on the topic consists of small and heterogeneous studies. No RCTs were found and while there is a systematic review it does not explicitly address the elderly population. Even if the target population was not specifically addressed, the published studies’ mean age ranges from 59 to 71 years. A recent systematic review analyzed functional outcomes after curative local excision of rectal cancer [122]. Of the available articles, 23 (79%) reported on pre-and postoperative fecal continence. Of them, 18 studies evaluated changes after transanal endoscopic microsurgery (TEM) and five after transanal minimally invasive surgery (TAMIS), with a mean follow-up of 15.9 months. Ten studies reported results using the Wexner score, seven used the Fecal Incontinence Severity Index (FISI), one study used the colorectal functional outcome (COREFO) questionnaire, one study used both Wexner and COREFO scores, and one study used an individualized interview. The remaining studies used the Kirwan-Fazio scale, the Williams score, and the Pescatori scale. Two studies found an increase in Wexner score after surgery (worsening of continence status) [123, 124], two studies found no changes in pre-and postoperative values [125–127], and one found a decrease in the Wexner score (better continence) [128]. When an alteration of anorectal function was reported in other studies, the effect on anorectal function was mostly transient, with restoration of functional status within a year from the operation. When compared with total mesorectal excision (TME), the patients who underwent local excision for rectal malignancies reported fewer defecation problems. Half of the retrieved articles reported improvement in Quality of Life (QoL) [125, 129–132], four remained comparable with preoperative values [133–136], and only one study had worsening in some QoL components [137]. Fifteen studies (51%) investigated manometric variables pre-and postoperatively. When manometry was used, no impairment or a transient impairment was observed, but not associated with a worsening of the continence status [123, 138].

There are five systematic reviews and meta-analyses that analyzed oncological results of local excision with curative intent of early rectal cancer, one comparing Transanal Excision (TAE) and TEM [139], four TEM and TME [140–143], and three RCTs [144–146]. Even in this case, rarely the considered studies mentioned a specific population. The mean age of the included patients ranged from 58.3 to 75 years. In one observational study, age was not considered a risk factor for recurrence [147]. A recent study [148] analyzed the oncological outcomes of 2.996 patients with pT1 and pT2 distal rectal adenocarcinomas who underwent local excision (1.795) and low anterior resection. The authors concluded that local excision is an acceptable oncologic treatment for T1 rectal cancer, while local excision with chemo-radiotherapy could be acceptable for T2 distal cancers. All the meta-analyses but one agreed that the oncological outcomes between local excision and TME are comparable in overall survival, five-year disease-free survival, and distant recurrence. Five-year disease-free survival reported by Kidane et al. using unadjusted risk ratios from 10 studies comparing local resection to radical resection ranged from 0.31 to 8.31 [140]. For the patients who received TAE (5 studies), the risk ratio ranged from 0.31 to 2.17, and for those who received TEM (5 studies), the risk ratio ranged from 0.49 to 8.31. The two RCTs comparing oncological results for early rectal cancer after local excision and TME found no differences in overall and five-year disease-free survival [144–146]. Chen et al. reported 100% overall survival for both groups at one year [145], while Winde et al. [144] reported 96% overall survival after a mean follow-up of 45.8 months in the radical resection group and 40.9 months after TEM. The meta-analysis by Kidane et al. [140] concluded that local excision does not offer oncologic control comparable to radical surgery. However, this finding might be driven by the higher prevalence of cancers with a poorer prognosis in local excision groups. Another meta-analysis comparing TEM, TME, and TAE concluded that while no survival advantage was observed in favor of either procedure, TEM had a lower rate of positive margins and more prolonged disease-free survival when compared with TAE [143]. An observational study [149] found that male gender, age, and surgical technique were significant risk factors for death after surgery in both univariate and multivariate analyses. An ongoing multicenter RCT, the TESAR, aims to determine the optimal treatment strategy for patients with a locally excised rectal lesion revealing an early stage rectal cancer with post excision pathology predicting intermediate (5–20%) risk of recurrence. The patients will be randomized, after local excision, to receive either adjuvant chemo-radiotherapy or standard completion TME surgery [150].

### Consensus Topic: E. Surgery

**Key Question 11.** Local excision. In elderly patients with a low invasive rectal cancer, how does local excision with palliative intent, if feasible, affect functional and oncological outcomes compared to rectal resection with TME?

**Statement.** Local excision is used in combination with neoadjuvant chemo-radiotherapy as an alternative tool to major resection in more advanced rectal cancer. Even if a study specifically addressing the elderly population does not currently exist, the mean age of patients undergoing such a management is higher than those receiving TME. In this case, anorectal function after excision may be affected by the radiation therapy but still seems to be better than in TME patients. Regarding oncological outcomes, there seems to be no difference between radical TME and local excision with palliative purposes.

**Recommendation.** The experts’ panel suggests to consider local excision as a palliative approach in elderly patients when they are judged unfit for major surgery, in combination with neoadjuvant therapy, when feasible (*Weak recommendation, Low quality of evidence—2C*).

**Agreement:** 91.2%

While local excision is generally considered a potentially curative procedure for early rectal cancer, its use with palliative intent has been rarely investigated in the current literature. To this end, several published series have analyzed mixed populations that underwent local excision for either palliative or curative intent, making it challenging to analyze the outcomes. A retrospective review published in 2001 compared the palliation achieved with endoscopic transanal resection (ETAR) with transabdominal resection [151]. Twenty-four patients who underwent local excision were matched with 25 patients who underwent palliative low anterior resection (LAR), Abdominoperineal resection (APR), or Hartmann’s procedure. Survival was similar in the two groups, and there was significantly higher morbidity in the group receiving open surgery, with a significantly higher stoma rate. The authors suggested that local excision may be considered a palliative option for low fixed rectal tumors that may be difficult to treat with LAR and for very elderly patients who may not be candidates for general anesthesia [151]. Since frail patients are more likely to experience postoperative complications, and the type of surgery is a determinant of postoperative adverse outcomes, the likelihood of confounding by indication needed to be accounted for [152]. Notably, the consequences of complications are far more severe and life-threatening in elderly patients than in younger patients. Rutten et al. [153], who assessed the data from a Dutch study on TME, showed in a comparative age analysis that older patients had increased 30-day and 6-month mortality and questioned how treatment-related mortality might obscure the oncological advantage of advanced surgical treatments in patients >75 years old.

A nationwide propensity score-matched study published in 2020 by Hoendervangers et al. [154] showed how patient and disease characteristics might influence patients’ selection for neoadjuvant and subsequent surgical treatment. The study involved 2.926 patients, and the primary goal was to investigate the effect of short-course radiotherapy delay on postoperative outcomes compared with standard chemo-radiation in both the general and the frail population. The study’s primary bias resides in the confounding indication of radiation therapy. The RCT by Lezoche et al. compared local excision following chemo-radiotherapy to TME in cT2N0M0 patients with rectal cancer, and it concluded that oncologic results were similar in the two groups with a median follow-up of 9.6 years [155]. The mean age of the enrolled patients was 66 years in both the study groups. A subsequent meta-analysis confirmed these results, finding no statistical difference in local recurrence, overall survival, and disease-free survival rates in patients who underwent local excision following chemo-radiotherapy or radical surgery for rectal cancer, despite the variability regarding the observational nature and the high heterogeneity in the selection criteria of most of the studies under consideration [156]. In the ACOSOG Z6041 trial, the estimated 3-year disease-free survival for the intention-to-treat group was 88.2% and for the per-protocol group (ypT0–2 tumors with negative margins) 86.9%; local recurrence rate was 4%, and 72 of 79 patients (91%) had rectal preservation [157]. In the GRECCAR 2 trial, patients were randomized in two study arms: the local resection group and the TME group [158]. The study concluded that there was no significant difference in local recurrence and disease-free survival at 3 years between the local excision group and the TME group (5% and 6% of local recurrence and 78% and 76%, respectively). In the CARTS study, the 5-year local recurrence rate was 7.7% with 5-year disease-free survival and overall survival rates of 81.6 and 82.8%, respectively, preserving the rectum in 64% of patients with cT1–3N0 tumor [159]. As for functional outcomes, transanal local excision following chemo-radiotherapy may involve a significant decrease in anal resting and squeeze pressures, rectal capacity, and sensitivity compromising fecal continence. The alteration of anorectal function seemed to be related to neoadjuvant chemo-radiotherapy since it has been demonstrated how chemo-radiotherapy could affect the overall morbidity rate after TEM in the early postoperative period. However, it does not seem to affect the continence status after one year from surgery [158–161]. Age and gender could be additional factors to influence anorectal function [134].

### Consensus Topic: E. Surgery

**Key Question 12.** Local Excision. In elderly patients with a cT2/T3 N0 low rectal cancer, how does radiotherapy followed by local excision compared to rectal resection with TME affect functional and oncological outcomes?

**Statement.** In elderly patients with a small cT2/T3 N0 low rectal cancer, radiotherapy followed by local excision in clinically good responders may offer no long-term difference in oncological outcomes compared to TME. In elderly patients with a cT2/T3 N0 low rectal cancer, radiotherapy followed by local excision may offer impaired functional outcomes, but in any case better than after TME.

**Recommendation.** The panel recommends to consider elderly patients with small cT2/T3 N0 low rectal cancers suitable for neoadjuvant therapy and organ sparing transanal local excision following chemo-radiotherapy (*Strong recommendation, Moderate quality of evidence—1B*).

**Agreement:** 91.2%

The elderly are generally less inclined to undergo major surgery, especially when there is a considerable risk either of a temporary or permanent stoma. They are, therefore, potentially good candidates for a less invasive transanal approach. To attain a curative intent, it was proposed 25 years ago [162] to perform TEM after neoadjuvant therapy. This generally includes both radio and chemotherapy. Radiotherapy may be administered both as long and short course.

Recently the data at 5-years of the first multicenter randomized phase 3 trial to compare organ preservation with radical surgery were made available [163]. The GRECCAR 2 study shows that in good clinical responders (i.e., when a > 50% reduction of the primitive tumor is observed), the rate of local recurrence, disease-free, and overall survival are comparable to the TME group. It has to be noticed that about $$ \raisebox{1ex}{$1$}\!\left/ \!\raisebox{-1ex}{$3$}\right. $$ of patients randomized for the local excision group received a TME for lack of sufficient local response. Therefore, the GRECCAR 2 study also advances the hypothesis that ypT2 cancers, mainly N0 at preoperative MRI, do not require a TME. These findings are in line with the ACOSOG phase 2 trial [157]. Similar results favoring chemo-radiotherapy and TEM were obtained after short-course radiotherapy in the TREC trial [164]. It is also known that the most extensive tumors have difficulties having an excellent response to chemo-radiotherapy, but this is today the object of the STAR TREC trial (NCT02945566) [165].

In a recent individual participant data pooled-analysis of published studies on rectal cancer surgery, logistic regression models were estimated for the risk of local, systemic and overall recurrence, showing a higher local and overall recurrences for ypT3 stage, tumor size after radiotherapy > 10 mm and lack of combined chemotherapy, while ypT3 was the only factor correlated with systemic recurrence [166].

Pucciarelli et al. [167] reported better overall Health-related quality of life (HRQL), constipation scores, and bowel function after local excision vs. TME following chemo-radiotherapy. Martens et al. [168] demonstrated that patients undergoing watch-and-wait procedures generally had good functional outcomes compared with seven patients undergoing TEM who experienced moderate outcomes. Major incontinence was seen in 42.8% of patients undergoing TEM. Finally, the CART study [159] analyzed long-term oncological outcomes and HRQL in patients with cT1-3N0M0 rectal cancer who underwent neoadjuvant CRT followed by TEM. HRQL during follow-up was equal to baseline, with improved emotional well-being in patients treated with local excision. Major, minor, and no low anterior resection syndrome was experienced in 50%, 28%, and 22%, respectively, in patients with successful organ preservation, confirming a reasonable rate of bowel dysfunction.

### Consensus Topic: E. Surgery

**Key Question 13.** Local Excision. In elderly patients who underwent local excision of a sessile polyp of the low rectum, with an unexpected result of a pT2/T3 Nx cancer on the resultant histopathology, how does postoperative radiotherapy compare to rectal resection with TME in terms of functional and oncological outcomes?

**Statement.** In elderly fit patients who underwent local excision for a low rectal sessile polyp with final pathology of pT2/T3 rectal cancer, radical surgery with TME is the treatment of choice. However, in case of contraindication to major surgery due to comorbidities, other treatments should be considered including adjuvant radiotherapy. The accurate definition of the surgical risk is a key point to guide towards the most appropriate decision.

**Recommendation.** The experts’ panel recommends radical surgery with TME as treatment of choice in elderly patients fit for surgery after the local excision of a sessile polyp of the low rectum subsequently confirmed as a pT2/T3 Nx cancer on the histopathology result (*Strong recommendation, Moderate quality of evidence—1B*).

**Agreement:** 96.8%

In the past, radical surgery with TME for rectal cancer in elderly patients has been questioned, suggesting more conservative approaches, including local excision combined with adjuvant (chemo)-radiotherapy even for T2-3 tumors [153].

However, new evidence demonstrated a similar life expectancy for patients undergoing rectal cancer surgery compared to the same age’s general population, even for patients > 80 years [8].

Two meta-analyses investigated oncological outcomes between different management after local excision for pT1–T2 rectal cancer. Van Oostendorp et al. compared no additional treatment (NAT), adjuvant (chemo)-radiotherapy (CRT) and completion TME (cTME): in 1.059 pT2 patients, local recurrence rate (LR) was 28.9% in NAT group, 14.7% in aCRT and 4% in cTME. Distant recurrence rate was respectively 6.2%, 5.8%, and 7% [169].

Borstlap et al. compared aCRT with cTME: in 341 pT2 patients, local recurrence was 15% in CRT the group and 10% in the cTME group [170].

These two meta-analyses have some limitations: no RCT was found comparing different treatments, and the included studies show heterogeneity regarding the local excision technique and CRT. Furthermore, the included studies are not specific for elderly patients.

A recent retrospective cohort study compared surgical outcomes between 10.631 patients (9.006 < 80 years and 1.625 > 80 years) with rectal cancer. Older patients showed higher ASA score than the younger counterpart (ASA 3: 52.4% vs. 25.4%; ASA 4: 6.4% vs. 2.1%; P < 0.001) and the rate of primary anastomosis was lower (75.5% vs. 83.6%; P < 0.001). There was no difference in overall surgical complications, but medical complications were higher in the older age group (25.2% vs. 11.2%; P < 0.001). Thirty-day mortality in the older group was higher than for patients < 80 years (3.1% vs. 0.4%; P < 0.001), but the multivariate analysis did not confirm any association between age alone and mortality rate [169]. We can recommend cTME after local excision of a pT2/T3 rectal cancer as the treatment of choice even in elderly patients, but it is crucial to assess, for an appropriate decision, specific risk, oncological risk, and life expectancy [8, 171].

### Consensus Topic: E. Surgery

**Key Question 14.** Minimally invasive surgery (laparoscopic/robotic TME, TaTME). In elderly patients with rectal cancer, how does minimally invasive surgery (laparoscopic/robotic-assisted) compared to open surgery affect recovery, functional and oncological outcomes?

**Statement.** In elderly fit patients with rectal cancer, a consistent amount of evidence suggests that laparoscopic TME is safe and feasible and is associated with short-term benefits compared to open surgery. There is insufficient evidence to support potential benefits of robotic and transanal approaches for rectal cancer resection in elderly patients compared to laparoscopy or open surgery.

**Recommendation.** The experts’ panel suggests laparoscopic TME in elderly fit patients with rectal cancer after a careful evaluation of patient’s medical history, performance status, and tumor characteristics (*Weak recommendation, Moderate quality of evidence—2B*). Minimally invasive surgery approaches other than laparoscopy and open surgery may be considered for TME in elderly patients with rectal cancer after a careful evaluation of patient’s medical history, performance status, and tumor characteristics. Open surgery may be appropriate in selected cases, including locally advanced tumors, multiple previous abdominal operations, or previous pelvic surgery. (*Neutral recommendation due to very limited and low-quality evidence*).

**Agreement:** 96.8%

Although short-term benefits and oncological adequacy of minimally invasive surgery (MIS), particularly laparoscopic resection, for rectal cancer is nowadays broadly investigated [172, 173], only limited evidence is available about the effectiveness of MIS approaches for elderly patients with rectal cancer.

Laparoscopic approaches to rectal cancer have increased over the past 30 years with laparoscopy providing shorter hospital stay, decreased postoperative pain, and faster return to normal daily activity compared to open surgery [174, 175].

Evidence for laparoscopic surgery for colorectal cancer in elderly patients suggests that laparoscopy is associated with fewer postoperative complications, shorter time to oral diet, and shorter hospital stay compared to open surgery, thus offering the same MIS-related advantages that are observed in patients of younger ages [176, 177]. When focusing on rectal cancer in octogenarian patients, a recent retrospective study demonstrated that laparoscopic rectal resection is as safe as open surgery. However, the known short-term advantages of laparoscopy may be lost in patients over 80 years due to a high rate of medical complications (40.4%), leaving open resection as an option in elderly patients with significant comorbidities [178].

Manceau et al. evaluated 446 consecutive rectal cancer patients grouping them into 10-year intervals from under 45 to older than 64 years. Elderly patients presented significantly higher ASA score, higher Charlson comorbidity index, and more frequent cardiovascular, pulmonary, and neurological comorbidities. Despite these baseline differences, there was no difference in postoperative complications and age was not a significant independent risk factor for postoperative morbidity [179]. Octogenarians rectal cancer patients were matched to controls between the ages of 60–69 in the study by Otsuka et al. They similarly found that the ASA score was significantly higher in the octogenarian group, but this did not correlate with increased postoperative complications and long-term rectal cancer-specific survival [180].

In the AlaCaRT trial, the primary end point was a composite of oncological factors (complete total mesorectal excision, a clear circumferential resection margin ≥ 1mm, a clear distal resection margin ≥ 1mm) indicating an adequate surgical resection, with a noninferiority boundary of Δ = − 8%. The primary outcome was achieved in 194 patients (82%) in the laparoscopic surgery group and 208 patients (89%) in the open surgery group, the circumferential resection margin was clear in 222 patients (93%) in the laparoscopic surgery group and in 228 patients (97%) in the open surgery group, the distal margin was clear in 236 patients (99%) in the laparoscopic surgery group and in 234 patients (99%) in the open surgery group, and total mesorectal excision was complete in 206 patients (87%) in the laparoscopic surgery group and 216 patients (92%) in the open surgery group. The study concluded that, among patients with T1–T3 rectal tumors, noninferiority of laparoscopic surgery compared with open surgery for successful resection was not established [181]. Similarly, in the ACOSOG Z6051 trial, the primary outcome of efficacy was a composite of circumferential radial margin ≥ 1 mm, distal margin without tumor, and completeness of total mesorectal excision. A 6% noninferiority margin was chosen according to clinical relevance estimation. Two hundred forty patients with laparoscopic resection and 222 with open resection were evaluable for analysis of the 486 enrolled. The primary outcome occurred in 81.7% of laparoscopic resection cases and 86.9% of open resection cases and did not support noninferiority [182]. However, subsequent analyses of the ACOSOG Z6051 trial found that laparoscopic-assisted resection of rectal cancer was not found to be significantly different to open resection of rectal cancer based on the outcomes of disease-free survival and recurrence. The 2-year disease-free survival was 79.5% in the laparoscopic group and 83.2% in the open group. Local and regional recurrence was 4.6% in the laparoscopic group and 4.5% in the open group. Distant recurrence was 14.6% in the laparoscopic group and 16.7% in the open group [183].

In general, elderly patients are considered at increased risk of postoperative morbidity and mortality and less likely to undergo laparoscopy or receive adjuvant chemotherapy after surgery compared with younger patients [184, 185].

Paucity of data on the oncological outcomes for laparoscopic versus open TME in the elderly has been recently remarked in the Bi-National Colorectal Cancer Cancer Audit (BCCA). MIS was performed in just over half of elderly rectal cancer patients who were selected for elective rectal resection surgery in Australia and New Zealand, but when performed in the elderly, MIS appeared safe and was associated with fewer wound complications and a shorter length of hospital stay at the propensity-score matched analysis, with comparable short-term oncological outcomes [186].

Several studies support that age is not a predictor of postoperative morbidity on its own, and rectal cancer resection can be safely performed by laparoscopy also in elderly (≥ 75 years) or very elderly (≥ 80 years) patients [179, 187–189]. Careful patient selection is advocated to choose the adequate surgical approach based on the patient’s performance status and tumor characteristics [179, 185, 187–189].

The majority of the available clinical data in elderly rectal cancer patients relates to the outcomes of laparoscopy compared to open surgery. Minimal evidence supports the use of robotic surgery, whereas TaTME remains essentially unexplored in the specific population of elderly patients. Some studies compared open vs. MIS surgery, which may include laparoscopy, robotic or transanal approaches with or without subgroup analyses describing the outcomes and advantages of a specific surgical technique.

The role of robotic surgery for colorectal cancer resection is still under investigation [190]. However, some evidence suggested that it may be associated with potential benefits over laparoscopy in terms of conversion rate, intraoperative blood loss, and hospital stay in general adult populations [191], which are also confirmed in studies analyzing rectal cancer patients only [192]. However, the evaluation of robotic surgery in elderly patients is rare. In a propensity score match study comparing robotic and laparoscopic colorectal cancer resections, de’Angelis et al. [193] showed that robotic surgery has similar operative and oncologic outcomes than laparoscopy in patients aged 70 years or more, despite longer operative times.

By examining the outcomes of robotic surgery versus laparoscopic surgery for rectal cancer based on evidence from 8 RCTs (1.305 patients), a recent systematic review and meta-analysis reported that age is positively associated with operative time and negatively associated with the length of hospital stay [194]. The longer operative time associated with robotic surgery might be seen as a clinically relevant disadvantage for elderly patients, but, surprisingly, a diminished trend of correlation has been observed as patients get older [194], meaning that in older patients the operative time difference between laparoscopic and robotic approach diminishes. Thus, these findings suggest that MIS should be preferred regardless of the patient’s age. Similar results were found in the most recent systematic review and meta-analysis comparing laparoscopic vs. robotic TME [195], which did not show any significant age difference and confirmed the downside of having longer operative times when performing robotic TME. This study also highlighted a decreased conversion rate to open surgery for robotic TME compared to laparoscopic TME and higher chances of being approached by MIS with robotic TME for patients with higher BMI, more distal rectal cancers, and after neoadjuvant treatments.

The NICE Guidelines [196] recommended laparoscopic surgery as the appropriate technique for most patients with surgically resectable rectal cancer. However, open surgery may be clinically indicated in locally advanced tumors or in patients with multiple previous abdominal operations or previous pelvic surgery (e.g., prostatectomy), which may likely be the case of elderly patients. NICE advised that robotic surgery should only be considered within established robotic programs and TaTME within structured and supervised programs, and data should be collected in a registry. Nonetheless, further studies are needed to confirm these promising results, and concern has been raised in both the UK and Norway in terms of oncological outcomes and complications in relation to TaTME [197].

### Consensus Topic: E. Surgery

**Key Question 15.** Early versus delayed ileostomy closure. In elderly patients with low rectal cancer who underwent low anterior resection with diverting loop ileostomy, how does early ileostomy closure compared to delayed ileostomy closure affect complications and quality of life?

**Statement.** In elderly patients with low rectal cancer who underwent low anterior resection with diverting loop ileostomy, early ileostomy closure is safe and feasible. Early closure is related with lower incidence of postoperative small bowel obstruction, stoma-related complications, and better functional outcomes, despite a relatively higher surgical site infection rate compared with late closure.

**Recommendation.** The experts’ panel suggests that in selected elderly fit patients, early (within 2 weeks) closure of ileostomy after rectal resection should be performed. (*Weak recommendation, Moderate quality of evidence—2B*).

**Agreement:** 87.9%

Anastomotic leakage (AL) represents a severe and common complication after rectal resection, whose incidence ranges between 2.0% and 10.3%, with peaks of up to 25% [198].

A study that focused on analyzing AL risk factors after anterior resection for rectal cancer in elderly patients over 80 years old found that the number of stapler firings ≥ three and coronary artery disease were independent risk factors for AL [199].

To decrease the severity of septic complications associated with AL in high-risk anastomoses and reduce the reoperation rate in the case of AL, a temporary diverting ileostomy (DI) is often performed at index surgery [200, 201].

The DI itself is associated with relevant morbidity, including skin irritation, parastomal hernias, stomal prolapse or retraction, and decreased quality of life (QoL) for the patient. DI-related morbidity rates reported in randomized controlled trials (RCTs) range from 2.9% to 62.2%, with a median rate of 14.3% [202]. As morbidity rates increase with time to ileostomy closure [203], it has been suggested that early closure (EC) of the DI could reduce adverse outcomes while still preserving the protective effect of the DI [204].

DI formation in higher risk elderly patients is independently associated with kidney injury, with an increased risk persisting after stoma closure [205]. Dehydration or renal failure following DI is common in elderly patients with metabolic disorders, leading to a 17% to 30% readmission rate [206]. Moreover, a DI during adjuvant chemotherapy is a predictor of severe chemotherapy-induced diarrhea and the need for modifications in the chemotherapy regimen. This may have significant consequences for long-term survival [207]. According to some authors, DI should be closed as early as possible because of their high morbidity, especially if adjuvant chemotherapy is planned [208].

There is no firm consensus as to the optimal timing of the closure of the DI, although the majority of centers classically choose to perform closure of DI at 8–12 weeks following rectal resection, once the integrity of the anastomosis is ensured [209]. To improve QoL, early stoma closure within 2 weeks from the index operation has been proposed. Six randomized controlled trials [204, 210–214] published over the past 10 years, and four systematic reviews with meta-analysis published over the past 2 years [202, 215–217] were evaluated by the meta-analysis team.

None of the studies performed sub-group analyses on clinical outcomes after early closure (EC) versus late closure (LC) in elderly patients, although both study groups included patients over 80 years of age. For this reason, the statement coming from the analysis performed in the general population has been downgraded due to some degree of indirectness and reported in this consensus on the elderly population with rectal cancer.

The meta-analysis by Wang et al. [215], that included four RCTs involving a total of 324 patients found that EC tended to result in more postoperative complications than LC for rectal cancer patients with DI (31.7% vs. 18.8%, RR = 1.70, P = 0.004), although the rate of severe complications was comparable (6.1% vs. 1%, RR = 4.41, P = 0.10). This difference was mainly influenced by the wound complication rate (20.4% vs. 9.7%, RR = 1.92, P = 0.07). LC resulted in more complications than EC before closure, such as leakage outside the appliance bag and skin irritation. The meta-analysis by Cheng et al. [216], that included a total of six RCTs, demonstrated that EC (within 2 weeks) of DI reduces the incidence of small bowel obstruction/postoperative ileus (3.0% vs. 7.8%, OR = 0.37, P = 0.01) and required shorter operative time (MD = − 9.68, P = 0.03), but increased the incidence of surgical site infection (11.3% vs. 3.6%, OR = 3.10, P = 0.004) compared with LC. Weak evidence showed that there was no difference between EC and LC in morbidity (20.1% vs. 20.0%, OR = 1.05, P = 0.84), reoperation (6.3% vs. 4.7%, OR = 1.40, P = 0.38) or leak of the rectal anastomosis (8.8% vs. 7.0%, OR = 1.28, P = 0.52) rates.

Ng et al. [217], in their pooled analysis of 667 patients from nine studies, confirmed the safety of EC, with an associated reduction in stoma-related complications (8.4% vs. 33.4%, RD = − 0.28, P = 0.001) despite a higher wound infection rate (18.6% vs. 7.1%, RD = 0.10, P = 0.047). The meta-analysis showed no significant difference in the postoperative morbidity rate, anastomotic leak rate, rates of small bowel obstruction, bleeding, and ileus between EC and LC. Also, the meta-analysis of six RCTs performed by Clausen et al. [202] could not discern a statistically significant difference in postoperative complications when comparing EC (within 2 weeks) and LC of DI. Overall postoperative morbidity in the EC group was 20.2% compared with 18.9% in the LC (RR = 1.13, P = 0.66), major complications (Clavien-Dindo grade ≥ 3) in the EC group was 5.2% compared with 3.6% in the LC group (RR = 1.12, P = 0.86), anastomotic leakage in the EC group was 3.3% compared with 3.5% in the LC group (RR = 0.89, P = 0.83). Reoperation rate was 5.9% in the EC group compared with 3.9% in the LC (RR = 1.35, P = 0.45). The authors performed a subgroup analysis of very early closure (defined as closure ≤ 3 weeks after index surgery) compared with late closure (closure > 6 weeks). The analysis did not provide a statistically significant difference between the two groups for any analyzed outcomes.

A meta-analysis that included four RCTs with 319 participants showed that the pursestring closure technique compared with the conventional primary closure group resulted in a significant decrease in surgical site infection (RD = − 0.25; P < 0.00001) [218, 219].

A substantial proportion of patients have severe bowel dysfunction many months after DI closure. A secondary analysis of the multicenter EASY (Early Closure of Temporary Ileostomy) RCT showed that patients undergoing EC had fewer problems with soiling and reduced risk of a permanent stoma, whereas patients undergoing LC have higher rates of bowel dysfunction, with an incidence of LARS (Low Anterior Resection Syndrome) of up to 73% of patients who had an ileostomy closed after 12 weeks [220]. However, although the EASY trial found that EC of the DI was associated with significantly fewer complications, this clinical advantage did not affect the patients’ health-related quality of life [221].

### Consensus Topic: F. Watch and wait

**Key Question 16.** Watch and wait, indications and outcomes. In elderly patients with rectal cancer, how does the watch and wait strategy in case of absence of clinically detectable residual tumor after neoadjuvant therapy affect functional and oncological outcomes compared to rectal resection?

**Statement.** In elderly patients with rectal cancer, in case of complete clinical response after neoadjuvant therapy, watch and wait may be considered a safe strategy, especially in selected patients, such as frail patients and patients with low-rectal tumors, with comparable oncological outcomes and better functional results in comparison to surgery.

**Recommendation.** The experts’ panel suggests a watch and wait strategy in selected frail elderly patients with low-rectal tumors in case of complete clinical response after neoadjuvant therapy. A stringent surveillance protocol, at least in the first 3 years, and a candid discussion with the patient about the potential risks of this strategy are recommended (*Weak recommendation, Low quality of evidence—2C*).

**Agreement:** 97.0%

The currently available literature is mainly observational, with no studies primarily focused on elderly populations. The first study by Habr-Gama et al. [222] in 2004 compared 71 patients who had complete clinical regression (cCR) after neoadjuvant chemo-radiotherapy undergoing watch and wait vs. 194 patients without complete response referred for surgery. The 5-year overall survival and disease-free survival rates were 100% and 92% in the watch and wait group vs. 88% and 83% in the surgery group. Overall recurrence and cancer-related mortality rates were 7.0% and 0% in the watch and wait group vs. 13.6% and 9% in the surgical group, respectively. Other single-center observational studies [223–226], albeit heterogeneous in diagnostic criteria and follow-up strategies, have confirmed similar results in terms of overall survival and disease-free survival in comparison to surgical resection with a follow-up ranging from 2 to 5 years. The first multi-institutional study on watch and wait strategies [227] included a cohort of 880 non-operatively managed patients from 47 institutions in Europe over a 25-year period with a median follow-up time of 3.3 years. Five-year overall survival and 5-year disease-specific survival were was 85% and 94%, respectively. The 2-year local regrowth rate was 25.2%, most of these (88%) were diagnosed in the first 2 years. Distant metastases were identified in 8% of cases. In a retrospective analysis of the same international registry [228], using a conditional survival model, the probability of remaining free from local recurrence for an additional 2 years, after a sustained clinical response, was estimated around 88.1%, 97.3%, and 98.6% at 1, 3, and 5 years respectively with analog results about the risk of distance metastatic disease highlighting the need of active surveillance especially in the first 3 years of follow-up.

A meta-analysis by Li et al. [229] comparing 251 patients with rectal cancer managed with a watch and wait approach vs. 344 patients that underwent surgical resection confirmed similar outcomes in terms of overall survival, disease-free survival, and distant metastasis rate but with a higher local recurrence rate at 1, 2, 3, and 5 years in the watch and wait group. These data were not confirmed in the meta-analysis by Dossa et al. [230]. This meta-analysis, published in 2020, aimed to quantify the additional risk of local recurrence for the watch and wait strategy, estimated a maximum additional risk of 6.5% at five years. However, the estimation was considered uncertain due to the high risk of bias in the current literature. Haak et al. [231] evaluated functional and oncological outcomes of patients aged ≥ 75 years from a collaborative Dutch database undergoing the watch and wait approach. Forty-three patients with at least 2 years of follow-up were included. The 3-year local recurrence-free rate was 88%, with a 3-year non-recurrence disease-free survival of 91% and overall survival of 97%. Functional outcomes (both defecations and urinary) at 3, 12, and 24 months were satisfying in most patients. Five patients (12%) had a local recurrence, but all underwent surgery with only one pelvic recurrence. Distant metastases occurred in 3 patients, and four patients died, but only one death was cancer-related.

In terms of functional outcomes, Maas et al. [223], in a prospective study of 21 patients with complete clinical response who underwent watch and wait vs. a control group of 20 patients submitted to surgical resection, reported more favorable functional outcomes with better quality of life and lower incontinence score, bowel function score, and mean defecation frequency in the watch and wait group. Smith et al. [232] used a model to compare three cohorts of men: 60-year-olds with mild comorbidities, 80-year-olds with minor comorbidities, and 80-year-olds with significant comorbidities (Charlson score > 3). Patients with a complete clinical response after chemo-radiotherapy were followed by a watch and wait protocol or offered radical surgery (TME). In both fit 80-year olds and those with comorbidities, there was a 10.1% survival advantage at one year in those who underwent a watch and wait approach. There were no differences between groups in disease-free survival or quality-adjusted life years. This model suggests elderly patients may have the most benefit from the watch and wait after a complete clinical response.

The current literature on watch and wait protocols in elderly patients is scarce and inadequate to formulate solid recommendations. Due to the absence of RCTs, standardized diagnostic and surveillance protocols, and the few studies tailored to the elderly population, the watch and wait strategy cannot replace surgery in the elderly. However, this approach may be offered as a safe option in high-risk patients, patients that would otherwise be potential candidates for abdominal-perineal resection, and patients refusing surgery, highlighting the potential higher risk of local recurrence and the need for a stricter follow-up in comparison with surgical therapy.

### Consensus Topic: G. Adjuvant chemotherapy

**Key Question 17.** Adjuvant chemotherapy. In elderly patients with rectal cancer who underwent radical surgery with curative intent, does fluoropyrimidine-based adjuvant chemotherapy improve the oncological outcome compared with clinical and radiological follow-up?

**Statement.** There is little evidence to support benefit of adjuvant chemotherapy for elderly patients with rectal cancer who have undergone radical surgery with curative intent compared with clinical and radiological follow-up.

**Recommendation.** The experts’ panel suggests that for selected stage III and stage II high-risk elderly patients with rectal cancer who underwent radical surgery with curative intent, a fluoropyrimidine-based adjuvant chemotherapy should be preferred to clinical and radiological follow up. Decision to perform adjuvant chemotherapy (alone or associated with radiotherapy) has to be taken after a multidimensional and geriatric assessment and must be shared within the multidisciplinary board, taking into account individual cancer risk of recurrence, DYPD evaluation, previous treatment (surgery alone or preoperative chemo-radiotherapy), patient’s performance status and comorbidities (*Weak recommendation, Low quality of evidence—2C*).

**Agreement:** 93.8%

According to the AIOM (Associazione Italiana di Oncologia Medica) ESMO (European Society for Medical Oncology) and NCCN (National Comprehensive Cancer Network) guidelines, adjuvant chemotherapy is recommended for patients with stage II/III rectal cancer, after preoperative chemo-radiotherapy and surgery [69, 233, 234].

Choice of adjuvant treatment regimen should be evaluated based on clinical and pathological risk factors, depending on both initial clinical staging and response to the preoperative treatment.

In those patients who underwent surgery without preoperative chemo-radiotherapy, adjuvant chemotherapy should be associated with radiotherapy treatment, leading to reduced local recurrence [235–238]. In contrast, RCTs and meta-analyses have failed to demonstrate a significant benefit for 5-FU chemotherapy alone as adjuvant treatment if a preoperative chemo-radiation strategy was performed [239–241].

Two recent RCTs suggest that adding oxaliplatin to 5-FU/leucovorin improves relapse-free survival and overall survival in high-risk rectal cancers [242, 243].

However, elderly patients with cancer are poorly represented in clinical trials constituting less than 10% of all the patients enrolled [244]. Consequently, most data on older patients with rectal cancer come from retrospective analyses and are often conflicting.

Results of a retrospective review of 286 patients suggest that patients with locally advanced rectal cancer who underwent surgery with curative intent (with or without preoperative therapy) gain a survival benefit from adjuvant 5-FU based chemotherapy, regardless of age [245].

These results are consistent with those from a large study by Xu et al., in which results from 14.742 patients with stage II and III rectal cancer from the U.S. National Cancer Database found that adjuvant 5-Fu based chemotherapy was an independent predictor of survival, regardless of patients’ factors, including age and comorbidity load [246].

The impact of age on clinical outcomes in patient with locally advanced rectal cancer receiving neoadjuvant chemo-radiation was also evaluated in a large multi-institutional retrospective review: the authors found that elderly patients > 70 years have similar outcomes compared with younger patients in term of disease-free survival, overall survival, and cancer-specific survival [247].

Many studies have shown that older patients are less likely to receive standard oncological treatments: reduced organ function and preexisting comorbidities can increase treatments’ toxicity that might contraindicate the use of chemotherapy [248–251].

To individualize the oncological therapies, a geriatric assessment is mandatory: current NCCN-ESMO guidelines suggest formal geriatric assessment before any treatment for patients over 70 years having cancer. Similarly, the International Society of Geriatric Oncology (SIOG) has recommended the use of systematic comprehensive cancer geriatric assessment [252]. In conclusion, retrospective population-based analyses and the absence of prospective randomized trials make it difficult to drive conclusions on the impact of adjuvant chemotherapy on oncological outcomes in elderly patients. Chronological age should not be a criterion for excluding standard treatment, and fit elderly patients should be treated according to standard guidelines. Multidimensional and geriatric assessments are mandatory, and decisions should be taken after multidisciplinary board discussion to evaluate risk-benefit balance.

Elderly patients with rectal cancer may benefit more from 3 vs. 6 months of adjuvant chemotherapy. For those with stage III rectal cancer (pT1–4, pN1–2, M0) treated with short-course radiotherapy or no preoperative treatment, capecitabine in combination with oxaliplatin (CAPOX) for 3 months or, if this is not suitable, oxaliplatin in combination with 5-fluorouracil and folinic acid (FOLFOX) for 3 to 6 months, might be considered a valid alternative to a 6-months single-agent fluoropyrimidine regimen [196].

### Consensus Topic: H. Liver disease

**Key Question 18.** Treatment of synchronous liver metastases: In elderly patients with rectal cancer, how do sequential resections (liver then rectum, or vice-versa) compared to simultaneous resection affect postoperative morbidity, mortality, and oncological outcomes?

**Statement.** Liver resections in elderly patients aged >75 years with colorectal liver metastases show equivalent disease-free survival compared with younger patients, although in these patients perioperative mortality is almost doubled and overall morbidity rate seems to be higher. Simultaneous and staged colorectal and hepatic resections for synchronous liver metastases have comparable postoperative morbidity and mortality, recurrence rate, and 5-year overall survival. However, the simultaneous approach seems to be safe only in selected elderly patients with less severe liver disease. Patients with a high burden of liver disease may be more likely to benefit from early liver-first approach after down-staging therapy.

**Recommendation.** The experts’ panel suggests staged or simultaneous liver resection for colorectal liver metastases in elderly patients depending on the burden of liver disease and patient’s frailty status. Caution should be taken in performing major hepatectomies in patients aged >75 years, given the increase in postoperative morbidity and mortality (*Weak recommendation, Moderate quality of evidence—2B*).

**Agreement:** 97.1%

In up to 25% of cases, colorectal cancer presents with simultaneous liver metastases, and 85% of these lesions are not resectable at diagnosis [253]. Liver resection is currently the treatment that offers the highest cure rate in patients with colorectal liver metastases (CRLM), with an overall 5- and 10-year survival rate ranging from 16 to 74% (median 38%) and 9 to 69% (median 26%), respectively [254]. The current literature on this issue lacks RCTs, whereas only retrospective studies with heterogeneous design, population, and outcomes are available.

In elderly patients, several observational studies have suggested that liver resection for CRLM is a safe treatment, with the 5-year survival rate reaching up to 40% [255]. However, treating elderly patients with CRLM is a challenge to date, as there is still a relevant lack of guidelines to support the decision of the optimal therapeutic strategy in these subgroups of patients.

The analysis of pooled data has shown that the weighted 5-year overall survival appears to be lower in patients aged > 70 years compared with their young counterparts (40 vs. 32%, P < 0.001), although the 5-year disease-free survival is comparable [256]. These results may justify a resectional approach in selected elderly patients with CRLM. On the other hand, the higher postoperative mortality rate found in elderly patients undergoing liver resection is more likely explained by the fact that this age population more frequently has coexisting chronic morbidity and has a more limited survival expectancy. The observation can support this assumption that the disease-free survival does not differ between the elderly and younger population in several systematic reviews and meta-analyses published to date, therefore suggesting that older patients die of different causes other than complications following the liver resection [185].

Several studies and meta-analyses compared both short-term and long-term outcomes in younger and elderly patients undergoing liver resection for CRLM. Elderly patients undergo significantly fewer major hepatectomies and are less likely to receive perioperative chemotherapy [185]. The systematic review and meta-analysis by van Tuil et al., with eleven studies that compared patients aged < 70 years with patients > 70 years and four studies that compared patients aged < 75 years with patients aged > 75 years, found that there were no significant differences in postoperative morbidity and 5-year disease-free survival for patients aged < 70 years and patients aged > 70 years, although postoperative morbidity and mortality both seem to be significantly higher in patients aged > 75 years. Postoperative morbidity was equivalent in patients aged >70 years (27 vs. 30%; P = 0.35), but higher in patients aged > 75 (21 vs. 32%; P = 0.001). Conversely, postoperative mortality was higher in both patient groups aged > 70 years (2 vs. 4%, P = 0.01) and in patients aged >75 years (1 vs. 6%, P = 0.02). In this meta-analysis, major hepatectomy was more frequently performed in patients aged < 75 years (61%) compared with patients aged > 75 years (53%) [256]. Similarly, de’Angelis et al., assessed the outcomes of 7579 older patients, 179 very old patients, and 15.904 young patients undergoing liver resection for CRLM in a pooled data analysis of postoperative outcomes, and showed that older patients were at 2 to 3-fold increased risk of postoperative mortality compared to younger patients [RR = 2.53] and found shorter overall survival [HR = 1.17] in older patients. However, no differences in operative outcomes, postoperative complications (bile leak, liver failure, pulmonary complications), and disease-free survival were found. Similarly, the occurrence of major postoperative complications (Clavien-Dindo III or more) was equivalent between older and younger patients. The majority of the studies included in the meta-analysis by de’Angelis et al. found that age was not an independent predictive factor of overall survival and disease-free survival, supporting the conclusion that advanced chronological age should not be regarded as a medical contraindication to liver resection for CRLM [185].

The ideal treatment strategy for colorectal cancer with synchronous CRLM remains a matter of debate due to the lack of RCTs and due to the rapidly changing systemic treatment modalities. The initial treatment is usually determined by the extent, resectability, and symptomatic burden of colorectal cancer and the concomitant metastatic liver load. Patients with rectal primaries have been shown to present most commonly with a higher metastatic liver load and undergoing a liver-first strategy following preoperative chemotherapy.

Four systematic reviews and meta-analyses comparing simultaneous and staged colorectal and liver resections for colorectal cancer with synchronous CRLM have been published over the last ten years, showing different strategies achieve nearly comparable outcomes.

According to the literature, patients with synchronous rectal cancer and liver metastases needing major hepatic resections are selected more frequently for staged operations. All the studies published to date on this topic show a significant selection bias associated with colorectal cancer site (right-sided vs. left-sided vs. rectum), the extension of liver resection, and use of neoadjuvant chemotherapy [257]. Hajibandeh et al. found that there was significantly lower use of neoadjuvant chemotherapy and minor hepatic resection in patients treated with a simultaneous colorectal and liver resection. No significant difference was found in postoperative morbidity (OR = 1.04), mortality (RD = 0.00), and 5-year overall survival (OR = 0.88) between the two strategies [258].

Although a network meta-analysis published in 2014 by Kelly et al. [259] found that the 5-year overall survival and the 30-day mortality rates did not show significant differences between the colorectal first, simultaneous, and liver-first approaches, an updated network meta-analysis on 44 retrospective studies reporting on 10.848 patients showed that, compared to the other two approaches, the simultaneous one resulted in a higher risk of major morbidity and 30-day mortality. From this analysis, it also appears that the liver-first approach is increasingly used in colorectal cancer patients with synchronous CRLM, specifically in those with rectal primaries and those with a high load of metastatic disease [260].

Recently, laparoscopy has been shown to confer better outcomes for liver resections compared with open surgery in older CRLM patients. Minimizing surgical trauma in this subgroup of frail patients can facilitate the patient’s recovery [261]. The comparison between laparoscopic and open surgery for liver resection in CRLM elderly patients performed by de’Angelis et al. showed that the operative approach is not a predictor of 5-year overall survival and 5-year disease-free survival. However, a significantly lower postoperative morbidity was found in association with laparoscopic hepatectomy, particularly in the age group < 80 years [185]. A systematic review and meta-analysis of high-quality studies demonstrated an unexpected survival benefit in favor of laparoscopic over open liver resection for CRLM in the long term [262]. A subgroup analysis exclusively focused on outcomes of elderly patients performed in the same study found that median survival was 53.1 months and 44.9 months in the laparoscopy compared to the open liver resection groups, with a longer 3-year average life expectancy among elderly patients with laparoscopically resected CRLM. Non-surgical local ablation therapies, such as radiofrequency ablation and radioembolization, can be selected in potentially resectable metastases only if patients have unfavorable performance status and/or severe comorbidities, or if patients refuse surgery.

### Consensus Topic: I. Emergency presentations

**Key Question 19.** Obstructing rectal cancer. In elderly patients with obstructing upper rectal cancer, how does bridge-to-surgery rectal stent placement compared to emergency surgery affect oncological outcomes and the rate of minimal access surgery?

**Statement.** In elderly patients with obstructing upper rectal cancer, bridge-to-surgery rectal stent placement (when possible) compared to emergency surgery could improve short-term results, even potentially increasing the rate of minimal access surgery, with similar disease-free and overall survival rates.

**Recommendation.** The experts’ panel suggests that in elderly patients with obstructing upper rectal cancer, bridge-to-surgery rectal stent placement (when possible) should be preferred over emergency surgery (*Weak recommendation, Moderate quality of evidence—2B*).

**Agreement:** 82.4%

Self-expandable metallic stent (SEMS) positioning is a well-recognized treatment for malignant colonic obstruction [263–265]. Nevertheless, SEMS placement for tumors close to the anal verge is difficult because of the probability of severe pain resulting from the proximity to the dentate line [266]. Moreover, although technological improvements increase the possibility of SEMS placement in the lower rectum [267], precise deployment of a stent for tumors close to the anal verge is technically tricky [268]. The premise relevant to the statement in question is that, although five systematic reviews and meta-analyses of randomized controlled trials have been published to date in the literature [269–273] that analyze bridge-to-surgery SEMS placement vs. emergency surgery in the treatment of obstructing left colon cancer, unfortunately, such systematic reviews show relevant limitations including the fact that they did not consider the elderly subpopulation in the analysis of the results, and that only a tiny proportion of the patients included in the RCTs were affected by rectal cancers. Therefore, the statement is the result of an extrapolation of evidence, albeit of medium-high level, not necessarily obtained on the reference population. Keeping these limitations in mind, bridge-to-surgery SEMS provides favorable short-term outcomes by reducing the overall complications and stoma formation in treating malignant left-sided colonic obstruction, although the 30-day mortality rate of SEMS is comparable to emergency surgery. Although SEMS could be associated with a higher incidence of systemic and overall recurrence rates in terms of long-term outcomes, both interventions have similar disease-free and overall survival rates. In the general population, the short-term advantages of bridge-to-surgery SEMS should be weighed against the potential long-term oncological hazards. However, it must be emphasized that, in an elderly population, especially over 80 years, the short-term advantages may be relatively more important than the long-term disadvantages.

SEMS is associated with lower short-term overall morbidity and lower rates of a temporary and permanent stoma in the literature. Arezzo et al. performed a systematic review and meta-analysis of RCTs comparing SEMS as bridge-to-surgery and emergency surgery for acute symptomatic malignant left-sided large bowel obstruction, having for primary outcome the overall morbidity within 60 days after surgery. This meta-analysis that included eight RCTs and 497 patients showed that overall morbidity within 60 days after surgery was 33.9% in SEMS-treated patients and 51.2% in ES-treated patients (RR = 0.59; P = 0.023). The temporary stoma rate was 33.9% after SEMS and 51.4% after ES (RR = 0.67; P < 0.001), while the permanent stoma rate was 22.2% after SEMS and 35.2% after ES (RR = 0.66; P = 0.003). Primary anastomosis was successful in 70.0% of SEMS-treated patients and 54.1% of ES-treated patients (RR = 1.29; P = 0.043) [269]. A systematic review of studies involving long-term tumor outcomes comparing SEMS with emergency surgery was conducted by Cao et al. Overall, the analysis of outcomes from 24 articles and 2.508 patients, including five RCTs, three prospective studies, and 16 retrospective studies showed that the 3-year survival rate (OR = 0.88, P = 0.05), 5-year survival rate (OR = 0.91, P = 0.67), 3-year disease-free survival rate (OR = 1.14, P = 0.65), 5-year disease-free survival rate (OR = 1.35, P = 0.17), overall recurrence rate (OR = 1.04, P = 0.14), and local recurrence rate (OR = 1.37, P = 0.92) were comparable between the two management strategies. Long-term survival results, including 5-year disease-free survival and overall survival, are equivalent between SEMS and emergency surgery [274, 275]. Regarding the safety profile of SEMS as bridge-to-surgery based on pathology, it has been shown that the presence of perineural invasion (RR = 0.58, P < 0.00001), lymphovascular invasion (RR = 0.68, P = 0.004) and vascular invasion (RR = 0.66, P = 0.04) in SEMS-treated patients were higher than those in patients treated with emergency surgery, although the difference in lymphatic invasion (RR = 0.92, 95% CI 0.77, 1.09, P = 0.33) in the meta-analysis by Hu et al. Conversely, the number of lymph nodes harvested in SEMS group was higher than that in the emergency surgery group (MD = − 3.18, P < 0.00001). The authors concluded that SEMS implantation in patients with acute malignant obstructive colorectal cancer might increase all those adverse tumor pathological characteristics that are mostly related to the poor prognosis of colorectal cancer. However, not only the adverse effect of SEMS on long-term survival has not been demonstrated, but also, and especially when patients are elderly, these aspects could be overshadowed by more important outcomes in this patient group, such as the quality of remaining life [276]. SEMS could also provide possible palliation for patients with bowel obstructions and unresectable colorectal cancer. Controlled trials that compared SEMS with surgical interventions as palliative treatments in unresectable obstructive colorectal cancer patients were analyzed by Takahashi et al. SEMS was shown to reduce the risk of early complications (OR = 0.34; P < 0.01), mortality (OR = 0.31; P < 0.01), and stoma creation (OR = 0.19; P < 0.01). However, SEMS placement was significantly associated with a higher risk of perforation of the large bowel in this pooled analysis (OR = 5.25; P < 0.01) and late complications (OR = 1.94; P = 0.03), it contributed significantly to better long-term survival (HR = 0.46; P < 0.01) [274].

## Conclusions

Although rectal cancer is predominantly a disease of older patients, current guidelines do not incorporate optimal treatment recommendations for the elderly and address only partially the associated specific challenges encountered in this population. The present 2021 SICG-SIFIPAC-SICE-WSES consensus for the multidisciplinary management of elderly patients with rectal cancer summarizes the results of an extensive analysis of the consistent evidence about the multidisciplinary management of elderly patients.

We recommend the adoption of strategies for patient involvement in healthcare decision-making, the evaluation of the social background, and a discussion with the patient about therapeutic modalities for rectal cancer. We also recommend against colorectal cancer screening in patients older than 85 years, whereas a careful selection on an individual basis for patients between the ages of 76 and 85 years, according to their health status, is advisable.

The decision to pursue or withhold radical surgery requires estimation not only regarding individual perioperative mortality, but also life expectancy, healthcare priorities, and the patient’s primary goals, such as prolongation of life versus maintenance of independence and symptom relief.

For the fit elderly patient with acceptable sphincter tone, standard of care therapy should be pursued, whereas frail patients with more advanced disease could benefit from local excision as a palliative approach in combination with neoadjuvant therapy, or more intensive radiotherapy options. For elderly patients who retain a good physical and mental condition, treatment that is given to younger patients is deemed appropriate, whereas for those with diminished physiological reserves and comorbid conditions, alternative treatments that keep surgical trauma to a minimum.

From this perspective, properly selected elderly patients with rectal cancer should be always considered for surgical resection. We suggest laparoscopic TME after a careful evaluation of patient’s medical history, performance status, and tumor characteristics. Conversely, local excision can be implemented when balancing frailty, oncological outcomes, functional outcomes, and life expectancy. A watch and wait strategy can be considered in selected frail elderly patients with low-rectal tumors in case of complete clinical response after neoadjuvant therapy. In these cases, we suggest a stringent surveillance protocol, at least in the first 3 years, and a candid discussion with the patient about the potential risks of this strategy is recommended. The above recommendations have been made based on the best available evidence to help to maximize rectal cancer therapeutic strategies while minimizing adverse impacts on functional outcomes and quality of life for these patients.

## Data Availability

Not Applicable.

## Abbreviations

ACS: American Cancer Society
AGA: American Gastroenterology Association
AIOM: Associazione Italiana di Oncologia Medica
AL: Anastomotic leak
APR: Abdominoperineal resection
ASGE: American Society of Gastrointestinal Endoscopy
BCCA: Bi-National Colorectal Cancer Audit
cCR: Clinical complete response
COREFO: Colorectal functional outcome
CRLM: Colorectal liver metastases
CT: Computed tomography
DI: Diverting ileostomy
EC: Early closure
EORTC: European Organisation for Research and Treatment of Cancer
ERAS: Enhanced recovery after surgery
ES: Emergency surgery
ESMO: European Society for Medical Oncology
ETAR: Endoscopic transanal resection
EURECCA: European Registration of Cancer Care
EUS: Endoscopic ultrasonography
FISI: Fecal Incontinence Severity Index
GRADE: Grading of Recommendations, Assessment, Development and Evaluation
HAIs: Hospital-acquired infections
HR: Hazard ratio
HRQL: Health-related quality of life
ICHOM: International Consortium for Health Outcomes Measurement
LAR: Low anterior resection
LARS: Low anterior resection syndrome
MBP: Mechanical bowel preparation
MIS: Minimally invasive surgery
MD: Mean difference
MRI: Magnetic resonance imaging
NAT: Non-adjuvant treatments
NCCN: National Comprehensive Cancer Network
nCRT: Neoadjuvant chemo-radiotherapy
NICE: National Institute for Health and Care Excellence
n-RCS: Non-randomized controlled study
OAP: Oral antibiotic prophylaxis
OR: Odds ratio
pCR: Pathology complete response
PLCCRT: Preoperative long-course chemo-radiotherapy
PRISMA: Preferred Reporting Items for Systematic Reviews and Meta-Analyses
PROMs: Patient-reported outcome measures
PSCRT: Preoperative short-course radiotherapy
QLQ-C30: Quality-of-Life Questionnaire-C30
QoE: Quality of evidence
QoL: Quality of life
RCT: Randomized controlled trial
RD: Risk difference
RR: Risk ratio
SEMS: Self-expanding metal stent
SICE: Società Italiana di Chirurgia Endoscopica e nuove tecnologie
SICG: Società Italiana di Chirurgia Geriatrica
SIFIPAC: Società Italiana di Fisiopatologia Chirurgica
SIOG: International Society of Geriatric Oncology
SoR: Strength of recommendation
SSI: Surgical-site infection
TAE: Transanal excision
TaTME: Transanal total mesorectal excision
TAMIS: Transanal minimally invasive surgery
TEM: Transanal endoscopic microsurgery
TME: Total mesorectal excision
TNT: Total neoadjuvant therapy
USD: United States dollars
USPSTF: U.S. Preventive Services Task Force
WHO: World Health Organization
WSES: World Society of Emergency Surgery
